# A Polycomb Group Protein Is Retained at Specific Sites on Chromatin in Mitosis

**DOI:** 10.1371/journal.pgen.1003135

**Published:** 2012-12-20

**Authors:** Nicole E. Follmer, Ajazul H. Wani, Nicole J. Francis

**Affiliations:** Department of Molecular and Cellular Biology, Harvard University, Cambridge, Massachusetts, United States of America; University of California San Francisco, United States of America

## Abstract

Epigenetic regulation of gene expression, including by Polycomb Group (PcG) proteins, may depend on heritable chromatin states, but how these states can be propagated through mitosis is unclear. Using immunofluorescence and biochemical fractionation, we find PcG proteins associated with mitotic chromosomes in *Drosophila* S2 cells. Genome-wide sequencing of chromatin immunoprecipitations (ChIP–SEQ) from mitotic cells indicates that Posterior Sex Combs (PSC) is not present at well-characterized PcG targets including *Hox* genes in mitosis, but does remain at a subset of interphase sites. Many of these persistent sites overlap with chromatin domain borders described by Sexton *et al.* (2012), which are genomic regions characterized by low levels of long range contacts. Persistent PSC binding sites flank both *Hox* gene clusters. We hypothesize that disruption of long-range chromatin contacts in mitosis contributes to PcG protein release from most sites, while persistent binding at sites with minimal long-range contacts may nucleate re-establishment of PcG binding and chromosome organization after mitosis.

## Author Summary

Gene expression profiles must be maintained through the cell cycle in many situations during development. How gene expression profiles are maintained through mitosis by transcriptional regulators like the Polycomb Group (PcG) proteins is not well understood. Here we find that PcG proteins remain associated with mitotic chromatin, and a small subset of PcG binding sites throughout the genome is maintained between interphase and mitosis. These persistent binding sites preferentially overlap borders of chromatin domains. These results suggest a model in which PcG proteins retained at border sites may nucleate re-binding of PcG protein within domains after mitosis.

## Introduction

Epigenetic mechanisms, including those used by the essential PcG proteins, mediate stable inheritance of gene expression patterns through mitotic divisions. During mitosis, chromosomes undergo dramatic structural and biochemical changes and transcription is repressed. Binding of many transcription factors and chromatin regulators is disrupted in mitosis through post-translational modification of the proteins or their chromatin substrate [1]–[3]. Some transcription factors and chromatin proteins have been shown to persist on mitotic chromosomes to facilitate reactivation or prevent derepression of genes in G1, in a phenomenon termed “mitotic bookmarking” [4]–[9]. In most cases, however, how gene regulatory information is preserved through mitosis is not understood.

PcG proteins are required to maintain gene silencing during development and in differentiated cells (reviewed in [10]–[15]). These proteins assemble into multiprotein complexes with an array of enzymatic and structural effects on chromatin (for detailed reviews on the biochemistry of PcG proteins, see [16], [17]). Genes regulated by the PcG thus likely have unique chromatin features including histone and protein modifications, tightly bound PcG proteins, and a locally altered chromatin structure. In *Drosophila*, Polycomb Response Elements (PREs), functional binding sites for PcG proteins [18], also participate in long range interactions, which are disrupted when PcG proteins are depleted [19], [20]. Long range interactions are influenced by insulator sequences, which are found near many well-studied PREs [21]–[24]. Insulator sequences restrict enhancer-promoter interactions, and delineate chromatin loops and large-scale domains [25]. Insulators function by binding several proteins, including CTCF, BEAF, Su(HW), Mod(mgd4) and CP190 [25]. The status of long range interactions in mitosis is not known.

The extensive biochemical characterization of PcG proteins has not yet elucidated how regulation by these proteins can be maintained through mitosis. One model is that PcG proteins remain bound to mitotic chromosomes. An alternative model is that most PcG proteins are released from mitotic chromosomes but certain proteins or chromatin features mark their binding sites through mitosis to direct re-establishment of PcG protein binding after mitosis [26]. In *Drosophila*, immunofluorescence and live cell imaging studies have provided evidence for loss of PcG proteins from mitotic chromosomes, and, in some cases, for retention of some PcG proteins [27]–[30]. Here, we use immunofluorescence, biochemical fractionation and ChIP to analyze PcG protein localization to chromatin in mitosis in *Drosophila* S2 cells. We describe persistent association of PcG proteins with mitotic chromosomes but loss at most specific binding sites. A class of PcG binding sites that persists in mitosis is described, which we hypothesize has unique functions in chromatin organization and heritable gene regulation.

## Results

### Polycomb Group proteins are not excluded from mitotic chromosomes

**Figure 1:**
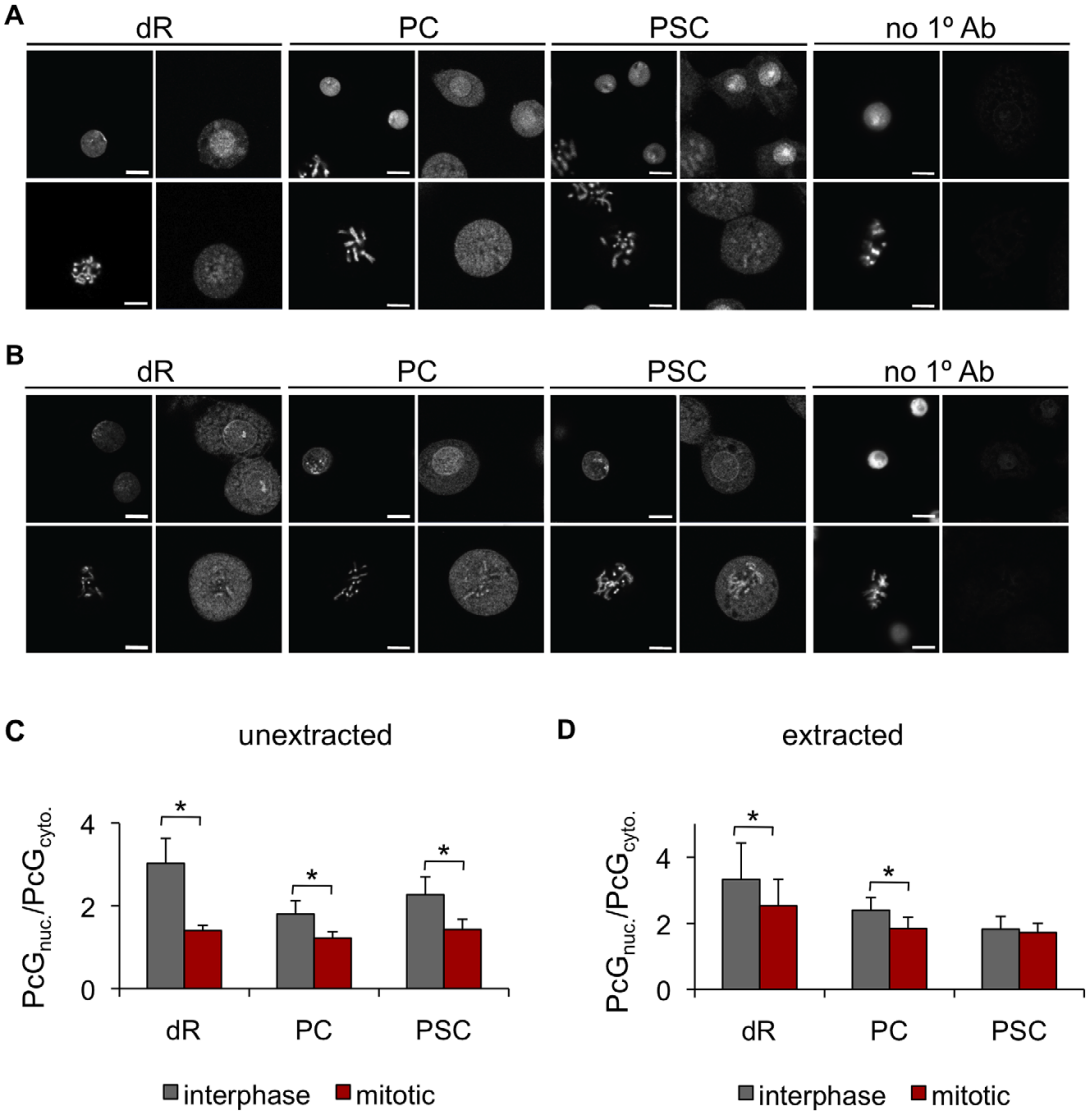
The PcG proteins PSC, PC, and dR are not excluded from mitotic chromosomes. A) Representative immunofluorescence images of *Drosophila* S2 cells stained with antibodies against dR, PC, PSC or no 1° antibody show dR, PC and PSC are not excluded from mitotic chromosomes. Left panels show Hoechst-stained DNA, and right panels immunofluorescence. Top rows are interphase cells and bottom rows are mitotic cells. B) Cells extracted with detergent prior to fixation and immunostaining do not show reduction in the fraction of the PcG signal that colocalizes with mitotic chromosomes. Panels are the same as A. Scale bar is 5 µm. C) Quantification of PcG signal that colocalizes with DNA versus PcG signal not on the DNA. n>10 cells for each category. All error bars show mean +/− S.D., in this and all other figures. * *P*<0.05 (two-tailed Student's *t*-test). D) Same as C for detergent-extracted cells.

We analyzed PcG protein localization to mitotic chromosomes in *Drosophila* S2 cells, a well-characterized cell line derived from embryos, by immunofluorescence. In interphase, Polycomb (PC), PSC, and dRING (dR) are predominantly nuclear, while in mitotic cells, they are distributed throughout the cell body, and neither restricted to nor excluded from chromosomes. Quantification confirms persistent but decreased signal for PcG proteins associated with chromatin in mitotic cells (Figure 1A, 1C). To ask if PcG proteins are more loosely associated with chromatin in mitotic cells, we extracted cells with detergent prior to fixation [31]. Detergent-extracted cells do not show reduced colocalization of PC or dR with mitotic chromosomes, and colocalization of PSC with chromatin is actually increased (Figure 1B, 1D), suggesting cytosolic PSC is extracted by the detergent. We conclude that PcG proteins are not excluded from mitotic chromosomes in S2 cells.

### Polycomb Group proteins fractionate with chromosomes in G2/M cells

**Figure 2:**
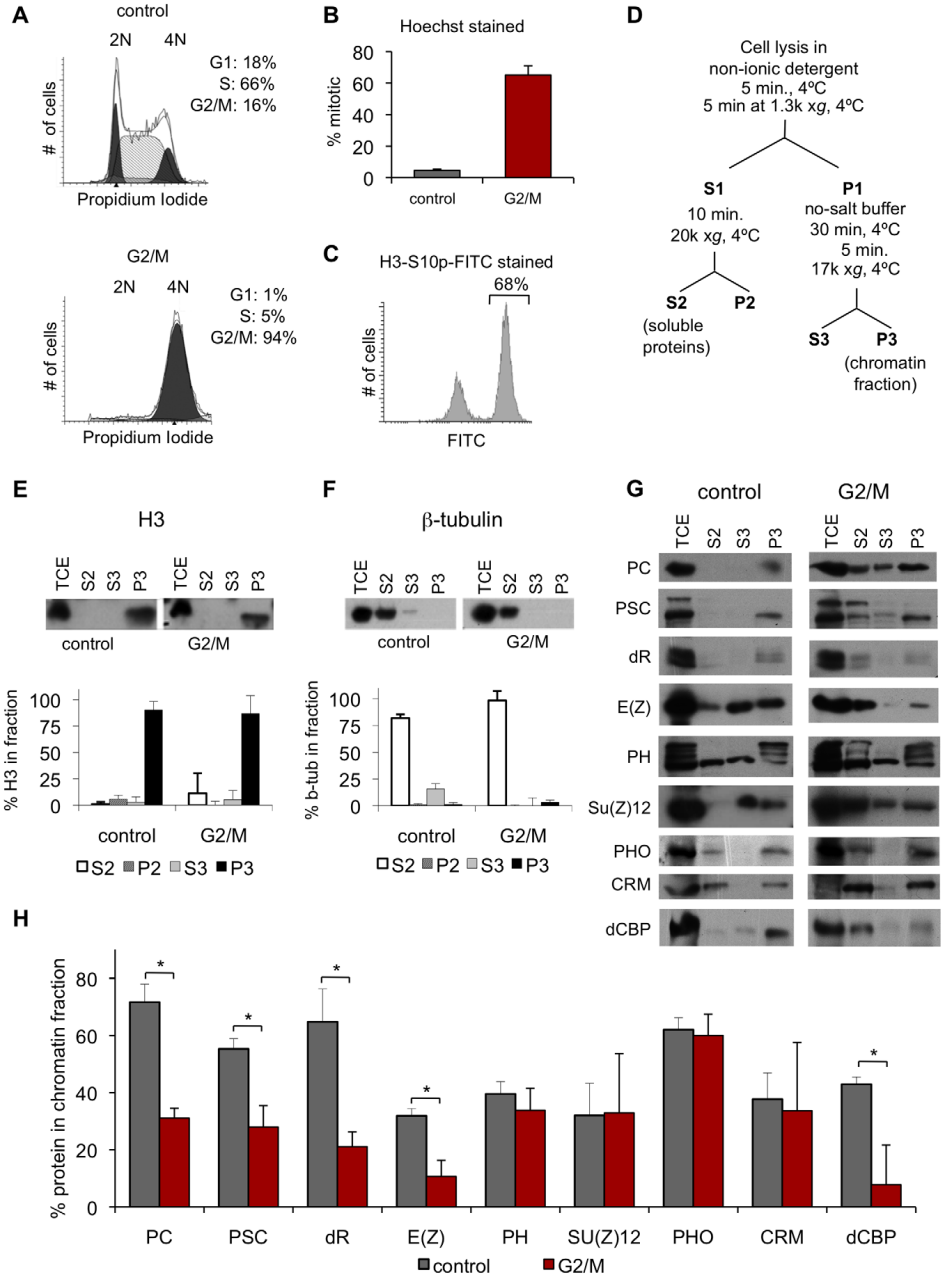
PcG proteins fractionate with mitotic chromosomes. A) Representative FACS profiles of propidium iodide stained cells, showing DNA content. Results of cell cycle analysis are shown in the upper right. Representative profile of a control culture (top panel) and a G2/M (colchicine treated) culture (bottom panel) shows that colchicine treated cells are ∼95% G2/M. B) Mitotic index of colchicine-treated G2/M cells determined by counting Hoechst-stained cells with condensed chromosomes shows that G2/M cells are 60–70% mitotic. C) Representative FACS profile of G2/M cells stained with FITC-conjugated α-H3S10p. Quantification of percentage FITC-positive is indicated and is in concordance with the mitotic index obtained by condensed chromosome counts. D) Schematic diagram of fractionation protocol used, adapted from [32] E, F) Representative western blot of fractions for histone H3 (E) or β-tubulin (F) (top panels) shows these proteins are present in the expected fractions. Quantification of the distribution of the protein in each fraction (bottom panels). G2/M samples were corrected for % of non-mitotic cells in the population according to: % P3_mitotic_ = [%P3-(%non-mitotic)*%P3_control_]/(%mitotic). *P<0.02 (two-tailed Student's *t*-test). G) Representative western blots of fractions for PcG proteins and dCBP for control and G2/M cells reveal that PcG proteins fractionate with mitotic chromatin. H) Graph of quantification of fraction P3 for multiple PcG proteins.

We used biochemical fractionation, followed by Western blot analysis, which does not depend on cell fixation or antigen accessibility, as an independent test of PcG protein association with mitotic chromosomes. To obtain the large amounts of mitotic cells needed for biochemical analysis, we treated *Drosophila* S2 cells with colchicine, a drug that blocks microtubule polymerization leading to metaphase arrest. At least 95% of colchicine treated cells have 4N DNA content, and about 66% of these are mitotic (Figure 2A–2C). We fractionated colchicine-treated (hereafter referred to as G2/M) and asynchronously growing (hereafter referred to as control) cells according to the scheme in Figure 2D, based on [32]. The distribution of several PcG proteins across the fractions was determined by Western blotting, and the percent of each protein in each fraction was quantified. For each set of G2/M cells, the mitotic index was measured (Figure 2B, 2C), and distributions of proteins were corrected to account for non-mitotic cells.

To validate the fractionation procedure, the distribution of β-tubulin and histone H3 was determined. β-tubulin is found primarily in the cytosolic fraction (S2), while H3 is found primarily in the chromatin pellet (P3), as expected (Figure 2E, 2F). In mitosis, the nuclear envelope is partially broken down which may allow mixing between nuclear and cytosolic proteins. Thus, the exact nature of S2 (cytosolic) and S3 (soluble nuclear) fractions in G2/M cells is unclear, although we expect that the S3 fraction will contain proteins that are loosely associated with chromatin in both control and G2/M cells. As a positive control, we analyzed the distribution of dCBP, a protein whose mammalian homolog has been reported to dissociate from mitotic chromosomes [33] (Figure 2G–2H, Table 1). The fraction of dCBP in the chromatin pellet (P3) in mitotic cells is 18% of that in control cells, consistent with dissociation of most of this protein in mitosis.

**Table 1:** Quantification of cellular fractionation.

		S2	P2	S3	P3
PC	control	6±2	1±1	22±6	72±6
	G2/M	45±5	2±2	21±1	31±3
PSC	control	25±2	2±0	18±4	55±4
	G2/M	59±11	4±1.0	9±7	28±7
dR	control	17±6	2±2	17±8	65±12
	G2/M	61±7	4±4	14±6	21±5
E(Z)	control	35±5	4±6	30±6	32±3
	G2/M	84±3	0±6	6±4	11±6
PH	control	30±2	3±1	27±3	40±4
	G2/M	49±2	0±4	17±3	34±8
SU(Z)12	control	30±9	2±1	37±7	32±11
	G2/M	50±23	7±4	10±7	33±21
PHO	control	25±5	8±3	5±5	62±4
	G2/M	38±16	0±1	2±8	60±7
CRM	control	60±9	1±1	1±1	38±9
	G2/M	65±26	0±1	1±2	34±24
dCBP	control	21±1	4±4	32±3	43±3
	G2/M	65±5	13±6	15±5	8±14

Normalized percent protein in each fraction.

We tested the distribution of 9 proteins, representing several PcG complexes. A large fraction of each PcG protein is in the chromatin pellet (P3) and soluble nuclear fraction (S3) in control cells, (Figure 2G–2H, Table 1). For all proteins, however, a portion is present in the cytosolic fraction (S2), which is consistent with the cytoplasmic staining we observe. In G2/M cells, a portion of each PcG protein fractionates with the chromatin, even after accounting for non-mitotic cells (Figure 2H, Table 1). For four proteins (PC, PSC, dR and E(Z)), the percentage of protein in the chromatin fraction is reduced in mitotic cells (61–71% of control) and increased in the cytosolic (S2) fraction. For the remaining proteins (PH, SU(Z)12, PHO and CRM) the fraction of protein in the chromatin pellet is nearly unchanged between control and mitotic cells (>85% of control). Biochemical fractionation data are consistent with immunofluorescence data that also show persistent but decreased PC, PSC, and dR associated with chromatin.

### PcG proteins are not detected at PREs in pure populations of mitotic cells

**Figure 3:**
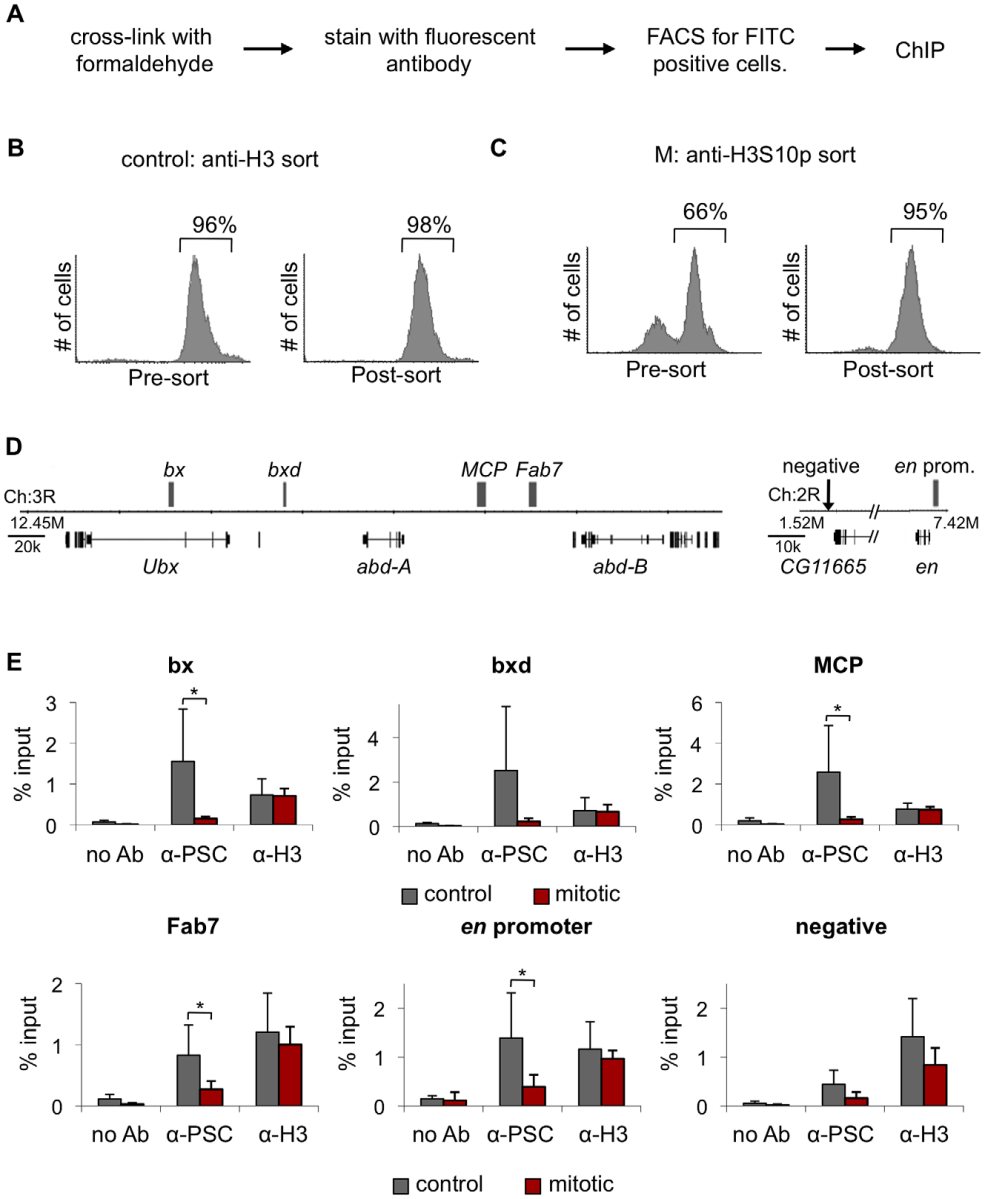
PSC binding is not detected at PREs in mitotic cells. A) Schematic diagram of the FACS sorting protocol to isolate mitotic cells based on H3S10p immunoreactivity. Control cultures were sorted with antibodies to histone H3. B) Representative FACS profiles of a control cell culture stained with α-H3 primary antibody and a FITC-conjugated secondary antibody before FACS sorting to isolate FITC-positive cells (left) and after sorting (right). Quantification of percentage FITC-positive cells is indicated and shows that post-sorted populations are ∼95% purely FITC-positive. C) Representative FACS profiles of G2/M cells stained with FITC-conjugated α-H3S10p antibody before (left) and after (right) sorting. Quantification of percentage FITC-positive cells is indicated and shows that post-sorted populations are ∼95% purely FITC-positive. D) Schematic diagram of part of the BX-C and the *engrailed* locus. Gray boxes indicates PREs. E) ChIP-qPCR for PSC and H3 in H3-sorted and H3S10p-sorted cells shows PSC binding is lost at these PREs in mitotic cells, while H3 binding remains the same between control and mitotic cells. *P<0.05 (two-tailed Student's *t*-test comparing mitotic and control).

Our analysis indicates that PcG proteins are associated with chromatin in mitotic cells but does not indicate whether they remain bound to target genes. To address this question, we used chromatin immunoprecipitation (ChIP). Pure populations (≥95%) of mitotic cells were isolated from colchicine-treated cultures using Fluorescence Activated Cell Sorting (FACS) with antibodies to histone H3 phosphorylated at serine 10 (H3S10p), which is a reliable marker of mitotic cells (Figure 3A–3C). To control for the FACS procedure, we sorted untreated cells with antibodies to histone H3. At least 95% of control H3-sorted cells are H3-FITC positive in the post-sorting analysis (Figure 3B). Starting with G2/M populations that are ∼66% mitotic, we obtain H3S10p-sorted cells that are ≥95% mitotic (Figure 3C). We used biotinylated antibodies to PSC and streptavidin-coated beads for ChIP-qPCR to avoid isolation of the antibodies used for sorting (and associated chromatin). To analyze the distribution of PH, we used a stable S2 cell line expressing low levels of biotinylated PH instead of antibodies (Figure S1A–S1C).

We analyzed several PcG binding sites within the Bithorax Complex (BX-C) of *Hox* genes: *bx, bxd, Fab-7* and *MCP* PREs, and two sites within the *engrailed (en)* locus (Figure 3D). PSC localizes to each of these sites in control, H3-sorted cells. In H3S10p-sorted, mitotic cells, however levels of PSC at these sites are indistinguishable from the level at a negative site (Figure 3E). In contrast, histone H3 is present at similar levels in mitotic and control cells. PH behaved similarly to PSC in a smaller number of experiments (not shown). We conclude that PSC is not detected at PREs in the BX-C and at the *en* locus in mitotic cells.

### Genome-wide binding profiles of PSC and PH reveal reduced chromatin binding in mitosis

To determine if PSC and PH are bound to any specific sites on mitotic chromosomes, we carried out ChIP-SEQ. Immunoprecipitated and input DNA from FACS-sorted mitotic and control cells was sequenced to generate genome-wide binding profiles for both PSC and PH. Between 4.9–16.3 million reads were uniquely mapped to the genome for each sample. 4,831 and 4,629 binding sites in control cells were identified for PSC and PH, respectively, using the MACS algorithm at a 5% false discovery rate (FDR) [34]. Two biological replicates of PSC binding profiles from control cells are in good agreement (Pearson's correlation coefficient, *r* = 0.97). We also find that the PSC and PH binding profiles are nearly identical, indicating a very high degree of colocalization for the two proteins (*r* = 0.96 (control); *r* = 0.97 (mitotic) and Figure S1D–S1F). A high degree of colocalization of PSC and PH has been observed by others by polytene chromosome staining and non-genome wide ChIP-chip [35], [36]. Another ChIP-SEQ study in S2 cells found almost complete colocalization of PH and PSC at PSC sites, but many PH only sites were also described [37]. We carried out only one ChIP-SEQ experiment with PH but included the data in our analysis because of the high overlap with our two biological replicates with PSC.

We compared our data for PSC with two published studies in S2 cells, a ChIP-chip study of PSC done by the modENCODE consortium, and a ChIP-SEQ study identifying overlapping sites for PSC, PC, PH, and TRX done by Enderle *et al.* PSC overlaps with 69% of PcG binding sites described by Enderle *et al.* and with 24% of the sites described by modENCODE (Table 2). The Enderle *et al.* dataset has 32% overlap with the modENCODE data. All three studies used different antibodies to the PSC protein; some of the differences in the datasets may be due to differences in sequencing vs. microarray technology [38]. Our PSC dataset also overlaps 27% of peaks in BG3 cells, and 56% of peaks from Kc cells identified in ChIP-chip experiments done by the modENCODE consortium [39], [40].

**Table 2:** Overlap of PSC binding sites with published datasets.

	# of sites	Overlap, modENDCODE PSC	Overlap, Enderle, *et al.* PcG
this study, PSC	4831	196/800 **(25%)**	1562/2274 **(69%)**
modENCODE PSC	800	-	278/2274 **(12%)**
Enderle, *et al.* PcG	2274	257/800 **(32%)**	-

**Figure 4:**
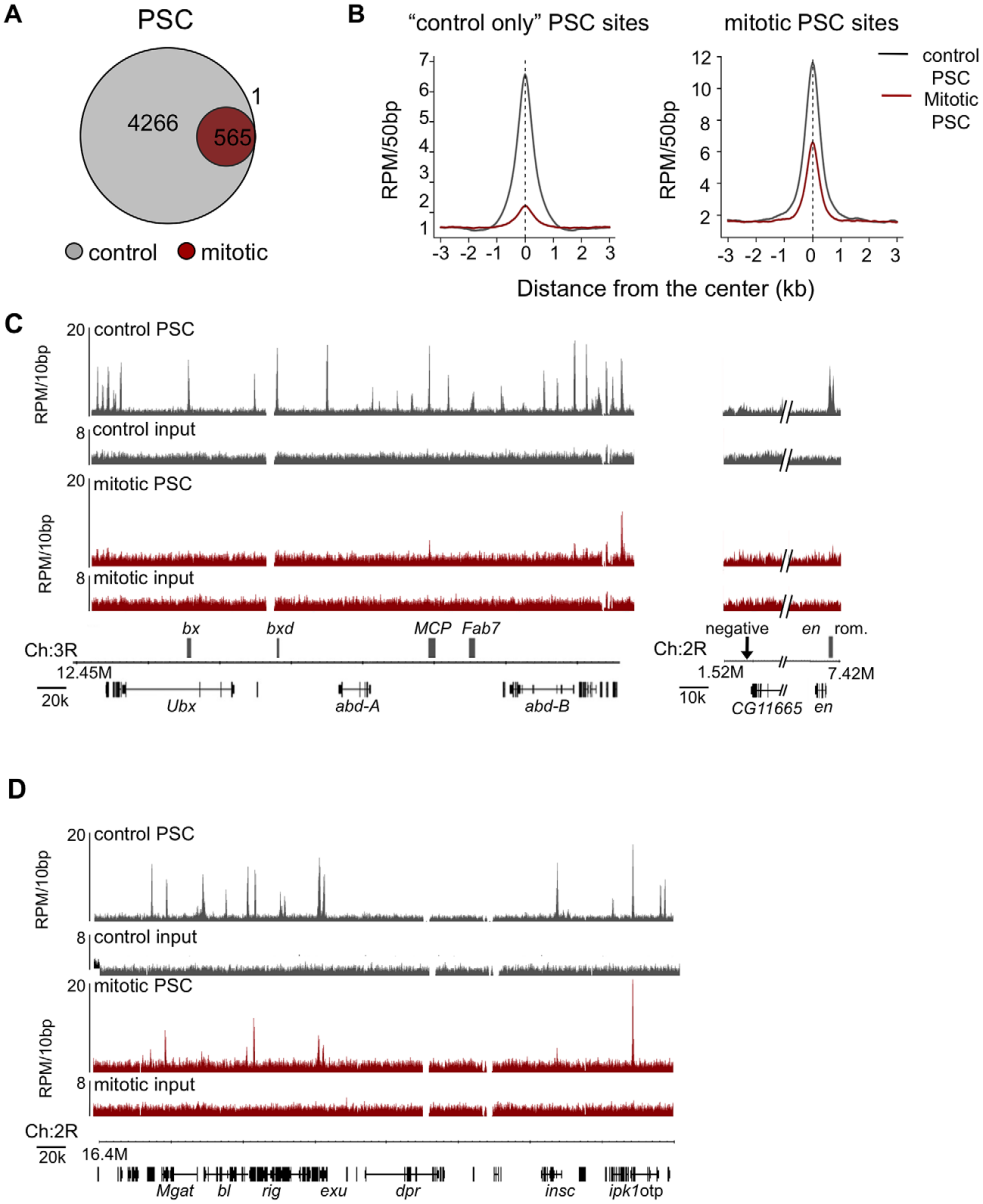
Genome-wide analysis reveals PSC is retained at specific sites on mitotic chromosomes. A) Venn diagram depicting overlap of peaks of PSC binding in H3-sorted (control) cells versus H3S10p-sorted (mitotic) cells shows that a subset of PSC binding sites are retained in mitosis and no new sites are bound in mitosis. Visual inspection of sequenced reads at the one peak exclusively in mitotic cells reveals it is likely not a PSC binding site. B) Normalized read density of PSC in control cells (gray line) and mitotic cells (red line) in 50 bp windows averaged over control-only PSC binding sites (left panel) or over all mitotic PSC binding sites (right panel) shows persistent yet reduced binding in mitosis genome-wide. C) Sequence tracks from ChIP-SEQ showing PSC binding control cells (top track, gray) and in mitotic cells (bottom track, red) over the BX-C and the *engrailed* locus confirm loss of binding at PREs within these loci in mitotic cells. PSC binding is lost globally across the BX-C. Y-axis is normalized reads per million (RPM)/10 bp. Chromosome position and gene models are shown at the bottom. D) Sequence tracks for PSC binding in control cells (top track, gray) and in mitotic S2 cells (bottom track, red) over a 400 kbp region of chromosome 2 show persistent mitotic binding sites. Y-axis is normalized reads per million (RPM)/10 bp. Chromosome position and gene models are shown at the bottom.

Using the same peak calling parameters as for control cells, we analyzed PSC and PH ChIP-SEQ data from mitotic cells. For PSC, we used an FDR of 5% for mitotic peaks, and identified 566 mitotic peaks, 39% of which overlap the modENCODE or Enderle datasets for PSC in asynchronous cells. For PH, the signal to noise in our profile from mitotic cells was lower than for PSC. Using a less stringent cutoff, 149 peaks were identified for PH in mitotic cells, 93% of which are also mitotic peaks of PSC. Due to the lower quality of this dataset, however, it was not included in subsequent analysis. The identified peaks for both PSC and PH are a subset of the peaks from control cells; no new peaks were identified in mitotic cells (Figure 4A). Average profiles of PSC in control and mitotic cells at “control only” sites (sites that do not persist in mitosis) confirm that sequenced reads are reduced at these sites in mitotic cells relative to control cells (Figure 4B, left panel). Average profiles of PSC in control and mitotic cells at “mitotic sites” (sites that persist in mitosis) reveals, on average, a reduction in sequence reads at these sites in mitosis (Figure 4B, right panel).

**Figure 5:**
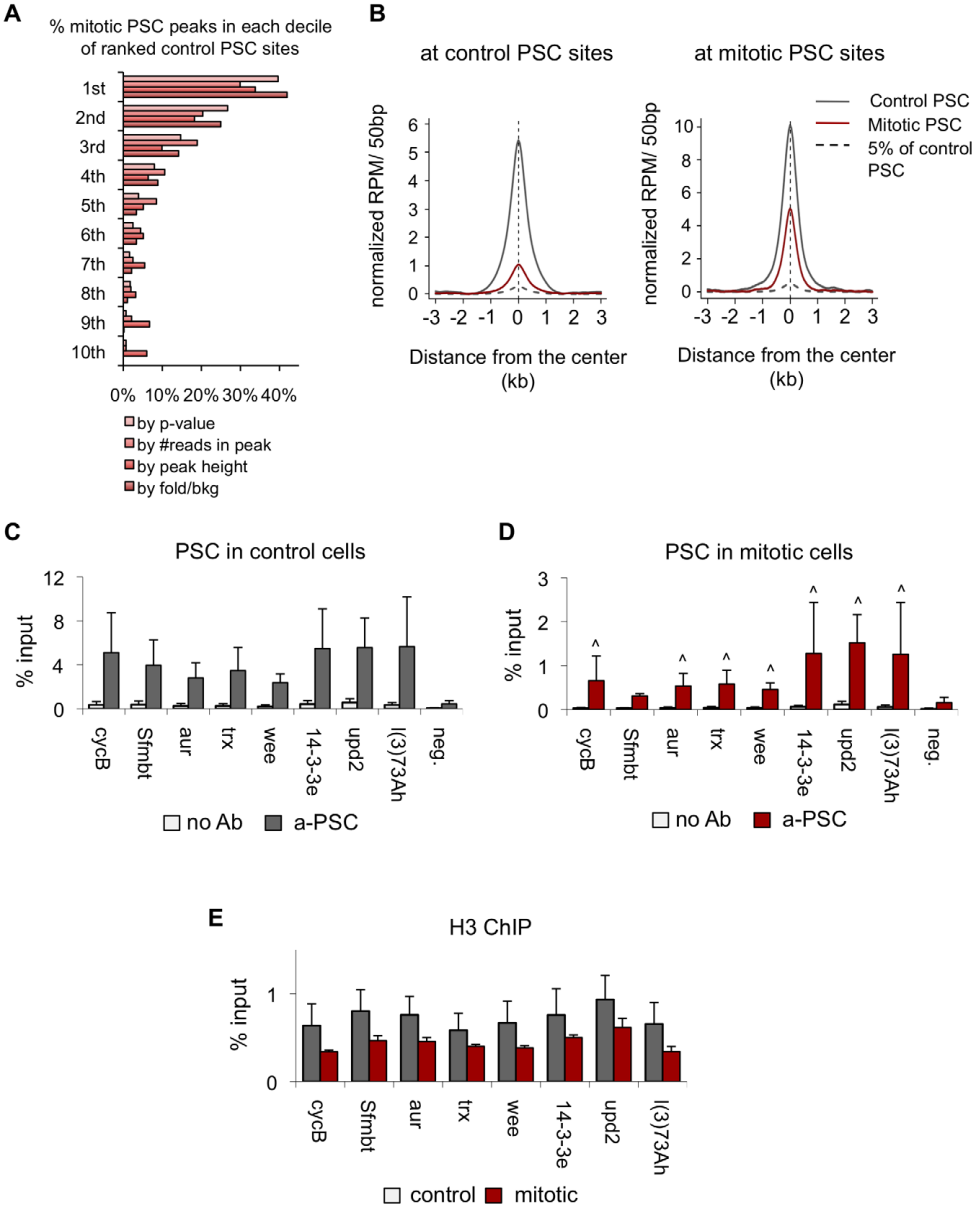
Validation of PSC peaks in control and mitotic cells. A) Plot of the percentage of mitotic PSC peaks per decile of corresponding ranked control PSC sites indicates mitotic peaks tend to be, but are not exclusively, the highest ranked peaks. Control PSC peaks are ranked by p-value, number of sequence reads per peak, peak height and fold over background. B) Average profile plot at control peak regions (left panel) or mitotic peak regions (right panel) for PSC in control cells (gray line), PSC in mitotic cells (red line) and 5% of the PSC profile in control cells in peak regions (gray dashed line) shows that the average plot for PSC in mitotic cells is greater than that for 5% of the PSC profile in control cells, indicating the mitotic peaks are likely not due to contamination of the mitotic population with up to 5% of interphase cells. C) ChIP-qPCR for PSC in control cells at eight binding sites identified by ChIP-SEQ and a negative site confirms PSC binding at the ChIP-SEQ identified sites but not the negative site in control cells. D) ChIP-qPCR for PSC in mitotic cells at eight binding sites identified by ChIP-SEQ and a negative site confirms PSC binding at the ChIP-SEQ identified sites but not the negative site in mitotic cells. ∧ *P*<0.05 (student's *t*-test). E) ChIP-qPCR for H3 in control and mitotic sites at eight binding sites identified by PSC ChIP-SEQ.

Examination of the PSC binding profile at the BX-C and *en* locus confirms the results observed by qPCR, which is that PSC binding is reduced in these regions in mitosis (Figure 4C). However, other peaks throughout the genome are clearly retained (Figure 4A, 4B, 4D). To determine whether the peaks that persist in mitosis are simply the largest peaks, we ranked control peaks by p-value, number of reads within the peak, peak height, or by fold over background and graphed the percentage of corresponding mitotic peaks per decile (Figure 5A). While the mitotic peaks tend to correspond to the higher ranked control peaks (30–40% of mitotic peaks correspond to peaks in the top decile of control peaks), they are not simply the highest ranked peaks. Instead, PSC is retained at specific sites. Visual inspection of the binding profiles also indicates that specific peaks are retained in mitotic cells despite the disappearance of neighboring peaks that are of similar size (Figure 4D). These data indicate that mitotic sites are unlikely to arise from contamination of the sorted cells with non-mitotic cells (up to 5%). To further validate this conclusion, we created an average profile using 5% of the control reads in peak regions and compared it with the total reads from mitotic cells (Figure 5B); the 5% control profile shows much less enrichment then the averaged mitotic profiles. We conclude that PSC is specifically retained at certain sites in mitosis.

We used qPCR to validate 8 peaks that are present in both control and mitotic cells (Figure 5C, 5D). We found that PSC is detectable at all of these sites in both control and mitotic cells but that the signals are lower in mitotic cells, consistent with the decreased number of reads. It is possible that the mitotic binding profiles observed reflect differing accessibility of chromatin in mitosis, rather than differences in PSC binding. To address this possibility, we compared ChIP for histone H3 in control and mitotic cells. At PREs, where the PSC ChIP signal is lost in mitotic cells, H3 signals are identical in control and mitotic cells (Figure 3D). At sites where PSC is retained in mitosis, we observe a slight decrease in the H3 ChIP signals, although these differences are not statistically significant (Figure 5E). These data argue against a general decrease in chromatin accessibility in mitotic cells, although we cannot completely exclude the possibility that access is differentially reduced for specific proteins at specific sites.

**Figure 6:**
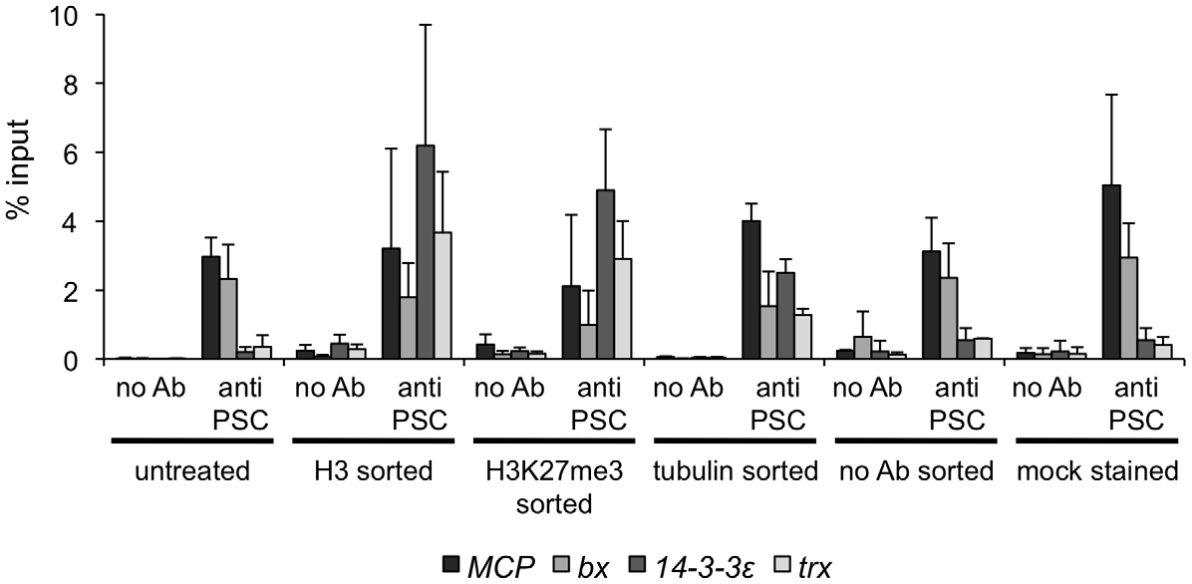
FACS sorting protocol increases chromatin accessibility. qPCR for PSC-ChIP from cells that were FACS sorted with one of three different antibodies, or subjected to only part of the sorting protocol (sorted without antibody or mock stained and not sorted). Note that PSC is detected at the two PREs, *MCP* and *bx*, under all conditions.

In our hands, the eight non-PRE sites tested by qPCR that are bound by PSC in both control and mitotic sorted cells are not detectable in asynchronously growing S2 cells that have not been sorted. Three of these eight sites were identified by Enderle *et al.* (2011) in their ChIP-SEQ analysis of unsorted S2 cells, however. This suggests many of the sites identified in our analysis, including some of those which are retained in mitosis, may be less accessible than well characterized PREs and thus may have distinct properties. Binding at these sites is specific for PSC, however, as no antibody ChIP controls give low signal at these sites. The detection of PSC at these sites is also not likely to be due to non-specific binding of the PSC antibody to these sites since biotin-tagged PH is detected at the same sites (i.e. without any ChIP antibodies). We carried out a series of control experiments with two of these sites to determine which aspect of the sorting procedure allows us to detect PSC at them (Figure 6). PSC was not detected at the *trx* or *14-3-3ε* genes in cells that were either subjected to the staining procedure (with or without inclusion of antibody), which involves incubation with detergent containing buffers, or FACS sorted without antibody. In contrast, binding at these sites is observed when cells are sorted with anti-H3K27me3, or anti-tubulin, although the signal from the tubulin sorted cells is reduced. Together, these results suggest the FACS procedure, and the antibodies used for sorting both contribute to detection of PSC at these sites. The exact cause of the increased accessibility is not yet clear. Two results are thus apparent from the genome wide analysis: 1) PSC is lost at a majority of its binding sites in mitosis; and 2) PSC is retained at specific sites.

### PSC is retained at sites across the genome and at cell cycle genes in mitosis

Because the mitotic binding sites for PSC did not include well-known target sites we analyzed them in several ways to understand their potential significance. Visual examination of the distribution of mitotic PSC sites over the chromosomes revealed that peaks are present on all chromosomes in control and mitotic cells, except for chromosome 4 where all peaks are lost in mitosis (Figure S2A). Quantification of the percentage of total sites per chromosome confirmed that the fraction of sites per chromosome remained relatively constant among the chromosomes between the mitotic and control binding sites except for chromosome 4 (Figure S2B). Analysis of the distribution of persistent sites within each chromosome arm suggests loss of sites from large regions along the chromosomes (Figure S2C) although the significance of this is not clear.

We mapped the genes in control and mitotic cells for which PSC lay within 2 kb of the transcription start site (TSS). The TSSs of 3,807 and 497 genes are bound by PSC in control and mitotic cells, respectively (mitotic site gene list, Table S1, and data not shown). We performed GO analysis on these gene lists using DAVID [41], [42]. As expected, many PSC-bound genes in both control and mitotic cells encode transcriptional regulators and those involved in development and differentiation (mitotic site, transcriptional regulator list, Table S2, and data not shown). Both gene lists are enriched for genes involved in the cell cycle. Interestingly, these cell cycle related genes are enriched in the PSC mitotic gene list when analyzed with the PSC control sites as background (mitotic site, cell cycle related gene list, Table S3) while genes encoding transcriptional regulators are not. The functional significance of this finding is unclear. Finally, PSC is retained at four PcG and seven Trithorax Group (TrxG) genes (PcG: *Asx*, *Sfmbt*, *E(Pc)*, *tan*; TrxG: *trx*, *osa*, *fs(1)h*, *E(bx)*, *utx*, *sbf*, *mod(mdg4)*, Table S1).

### PSC binding sites overlap with insulator proteins and chromatin domain borders

**Figure 7:**
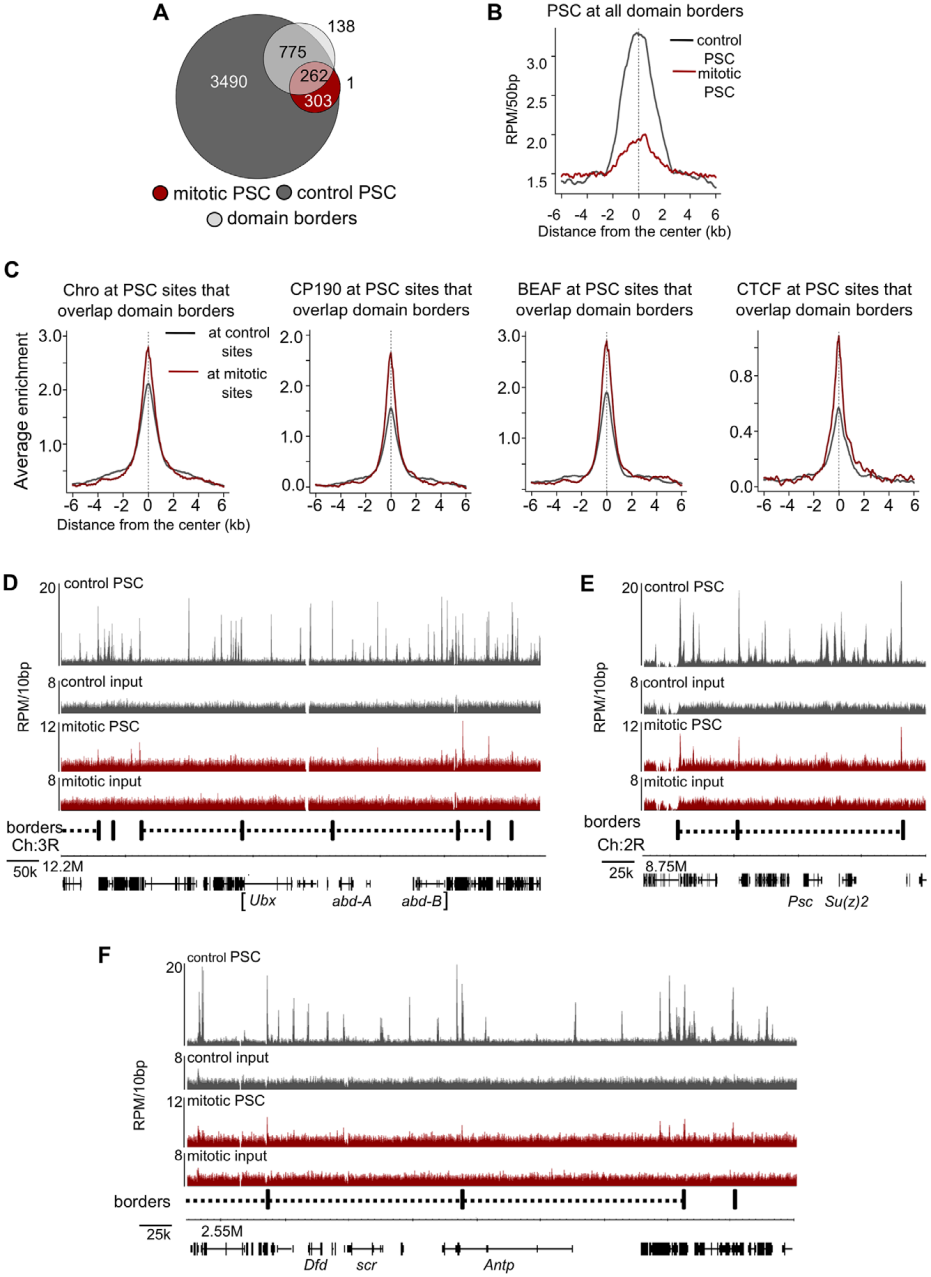
PSC preferentially binds domain borders in mitosis. A) Venn diagram showing overlap between domain borders and PSC binding sites in control and mitotic cells shows border sites are preferentially retained in mitotic cells. B) Average profile plot of PSC in control and mitotic cells surrounding domain borders shows enrichment of PSC at these sites. C) Average profile plot of Chromator (Chro), CP190, BEAF or CTCF surrounding PSC peaks at borders in control and mitotic sites shows enrichment of these proteins at PSC peaks at domain borders. D) Sequence tracks from ChIP-SEQ showing PSC binding in control and mitotic cells over the BX-C and surrounding regions show persistent PSC peaks at borders flanking the locus in mitotic cells. Domain borders are indicated as vertical black bars below the tracks. PcG domains identified by Sexton et al. (2012) [44] are indicated by dashed lines, and the BX-C is indicated by brackets below the gene models. (E,F) Sequence tracks from the *Psc*/*Su(z)2* locus (E) and ANT-C (F) showing PSC binding in control and mitotic cells in relation to borders reveal persistent PSC peaks at borders flanking these loci in mitotic cells.

**Figure 8:**
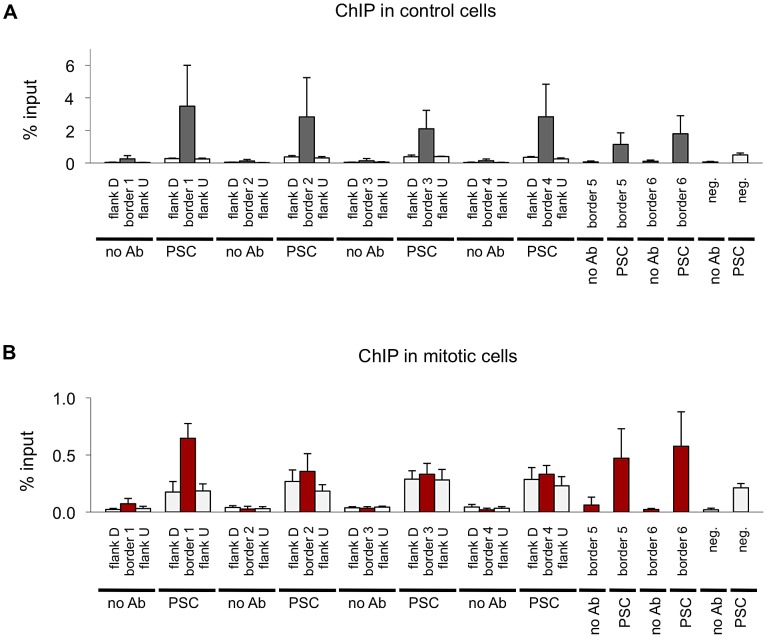
Validation of PSC binding a domain borders in control and mitotic cells. A) ChIP-qPCR for PSC in control sorted cells at six domain border sites and a negative site confirms PSC binding at the sites identified by ChIP-SEQ but not the negative site in control cells. Flanking sites are included for four of the border sites to confirm that binding detected by qPCR coincides with the peaks observed by ChIP-SEQ. B) ChIP-qPCR for PSC in control cells at six domain border sites and a negative site confirms PSC binding at the some of the ChIP-SEQ identified sites but not the negative site in control cells. Flanking sites are included for four of the border sites.

Next we compared the binding profiles of PSC from both control and mitotic cells with all chromatin-bound protein profiles from *Drosophila* S2 cells published by the modENCODE consortium [39] (not shown). Several proteins exhibited a high degree of overlap with binding profiles for PSC in both control and mitotic cells including the insulator proteins CP190, BEAF, and the mitotic spindle protein Chromator [43] (Table 3). Overlap with these proteins is higher for mitotic sites than total sites, suggesting overlapping sites are preferentially retained in mitosis. To confirm this overlap, we compared PSC peaks with additional datasets for CP190 and BEAF [43] (Table 3). These three proteins were recently identified as proteins that demarcate borders between physical and functional domains that exist in the *Drosophila* genome [44]. Mapping of physical contacts among chromosomal regions across the *Drosophila* genome revealed that chromosomes are partitioned into physical domains defined by their high intra-regional contacts. These domains correlate well with functional domains characterized by the binding profiles of various histone modifications and chromatin proteins within them. Borders are the regions between these domains, and, conversely to the physical domains, are identified by their paucity of long-range interactions. We find that PSC binds 88% of domain borders in control cells, which comprises 26% of total PSC binding sites (Figure 7A). Interestingly, 46% of all mitotic PSC sites overlap borders, indicating that these sites are preferentially retained in mitosis. 34% and 51% of the PSC peaks at border sites in control and mitotic cells, respectively, are PSC sites that have been previously described in the modENCODE or Enderle datasets. Average profiles from control and mitotic cells show enrichment of PSC at domain borders (Figure 7B). As expected Chromator, CP190, BEAF and CTCF are enriched at PSC peaks at domain borders in both mitotic and control cells, with greater enrichment on average at sites that are bound by PSC in mitotic cells (Figure 7C). We used qPCR to validate PSC binding at six border sites in control and mitotic cells (Figure 8). PSC is clearly bound to the border sites in control cells, while in mitotic cells PSC binding is reduced. The ChIP-qPCR signal at each of the border sites in mitosis is well above that of the no antibody control, yet at two of the border sites, binding is not higher than at flanking sites; we do not know why this is observed since it is not observed in the ChIP-SEQ traces. We conclude that one class of persistent binding sites for PSC overlaps borders of chromatin domains, which are also marked by Chromator and CP190, BEAF and CTCF.

**Table 3:** Overlap of mitotic PSC sites with published insulator datasets.

	# of sites	overlap mitotic PSC
CP190, modENCODE	6467	518/566 (92%)
CTCF, modENCODE	6227	499/566 (88%)
CTCF, Wood *et al.*	6691	513/566 (91%)
BEAF, modENCODE	4716	476/566 (84%)
BEAF, Wood *et al.*	6135	532/566 (94%)
Chromator, modENCODE	5319	494/566 (87%)

Re-examination of PSC binding at the BX-C in mitotic cells in the context of chromatin domains shows that while PSC binding is lost within the BX-C, it remains at the borders of PcG domains that encompass the cluster (Figure 7D). The same pattern was observed at the ANT-C and the *Psc*/*Su(z)2* complex (Figure 7E, 7F). Thus, at least some large domains of PcG protein binding are flanked by persistent peaks in mitosis. PcG binding sites within these clusters (none of which persist in mitosis) engage in extensive long-range interactions [20], [45], [46], [44], suggesting an inverse correlation between long range interactions and PcG protein persistence through mitosis.

### H3K27me3 persists at PREs in the BX-C and *en* gene in G2/M cells

**Figure 9:**
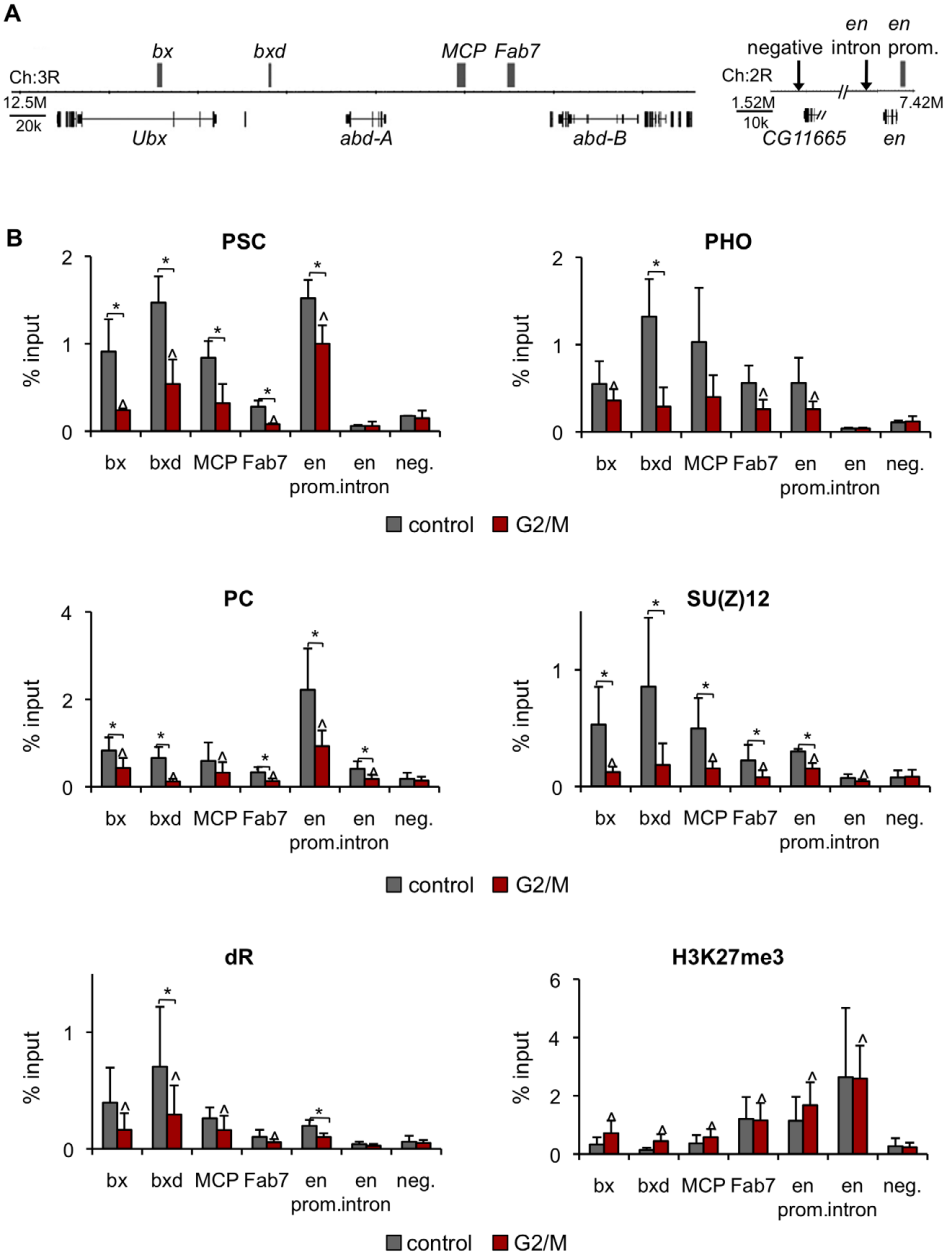
PcG protein binding, but not H3K27me3, is reduced at PREs in G2/M cells. A) Schematic diagram of part of the BX-C and the *en* locus. Gray boxes indicate PREs. B) ChIP-qPCR for PcG proteins and H3K27me3 in control and G2/M cultures is consistent with lost of PcG proteins but retention of H3K27me3 at these PREs in mitotic cells. *P<0.05 (two-tailed Student's *t*-test comparing control and G2/M). ∧ *P*<0.05 (two-tailed Student's *t*-test comparing G2/M to no antibody, not shown).

If PSC and PH binding is lost at target genes in mitosis, memory of repression may be carried by another PcG protein or the PcG-specific histone modification H3K27me3. To address this possibility ChIP assays were carried out on asynchronously growing (control) and colchicine treated (G2/M) S2 cells. PSC, PC, dR, SU(Z)12 binding and the PcG-specific histone modification H3K27me3 localization was analyzed for PREs in the BX-C and the *en* locus (Figure 9A). All PcG proteins and the H3K27me3 modification are bound at all target sites in control cells except for the *en* intron at which only PC and H3K27me3 are bound, but not at a negative site (Figure 9B). In G2/M cells PSC binding is reduced at PREs, consistent with our analysis of FACS sorted pure mitotic cells (compare Figure 9B to Figure 3E) and indicating that binding detected at PREs in G2/M cells is due to the presence of G2 cells in the population. Association of PHO, PC, SU(Z)12,and dR with PREs is reduced in G2/M cells similarly to PSC. Thus association of PHO, PC, SU(Z)12, and dR with these PREs may be lost in mitotic cells, although this will need to be confirmed with sorted mitotic cells. In contrast, the H3K27me3 modification is present at comparable levels at PREs in control and G2/M cells. S28 of histone H3 is phosphorylated in mitosis [47]. We do not know if the H3K27me3 antibody we used recognizes the H3K27me3/S28p double modification. It is therefore possible the level of H3K27me3 we see in G2/M cells is an underestimation, although we do not know if S28p is present on H3 at these sites. Nevertheless, our data indicates that the H3K27me3 modification most likely persists through mitosis, which is consistent with other reports [48]–[50].

## Discussion

**Figure 10:**
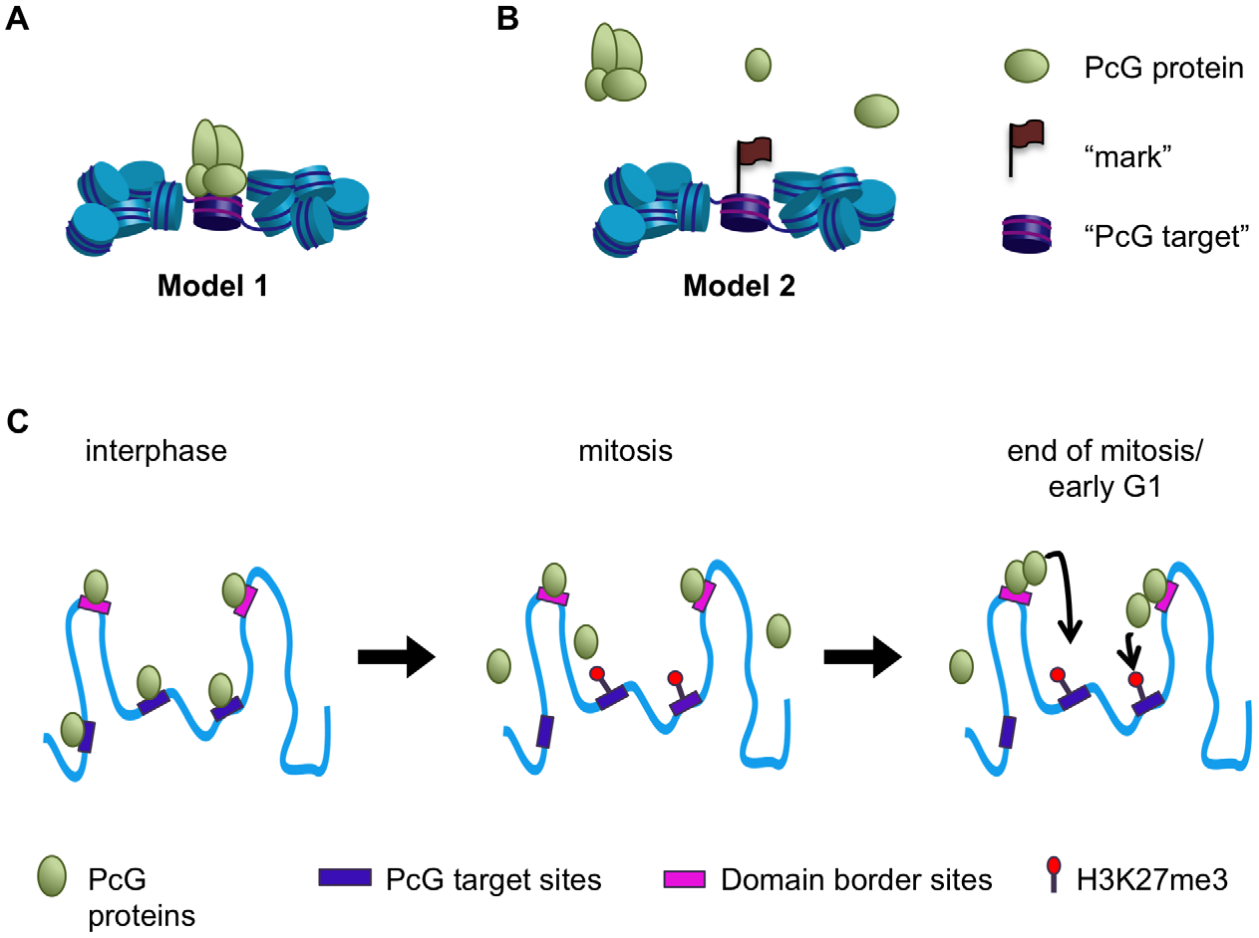
A model for maintenance of PcG protein function through mitosis. (A,B) Two models for the behavior of PcG proteins during mitosis. In Model 1 PcG proteins remain bound to mitotic chromosomes and may constitute memory of transcriptional repression through mitosis themselves (A). In Model 2 PcG proteins are released from mitotic chromosomes, but may leave a “mark”–a protein or chromatin feature—that persists through mitosis to allow rebinding upon mitotic exit (B). C) In interphase PcG proteins bind to specific target sites, including domain borders. During mitosis PcG proteins are lost from PREs and other previously well-characterized target sites but are retained at domain border sites. The histone modification H3K27me3 is likely retained at PREs in mitosis. At the end of mitosis or during early G1 PcG proteins retained at border sites may nucleate re-binding at PREs and other target sites within the domains, perhaps in conjunction with H3K27me3.

We set out to determine whether PcG-dependent repression might be propagated through mitosis in *Drosophila* cells by persistent binding of PcG proteins (Model 1, Figure 10A), or whether binding needs to be re-established after mitosis (Model 2, Figure 10B). Using three different methods (immunofluorescence, biochemical fractionation, and ChIP-SEQ), we show that PcG proteins are associated with mitotic chromosomes. ChIP-SEQ analysis, however, indicates that PSC and likely PH binding is retained at only a subset of interphase sites. Thus, the mitotic behavior of PcG proteins displays features of both Models 1 and 2, prompting consideration of new models (Figure 10C).

By immunofluorescence, we observe PSC, PC, and dR associated with mitotic chromosomes, although at reduced levels compared with interphase cells (Figure 1). These results are consistent with some studies [51], [28], and at odds with the conclusions of other investigators for PSC and PC [27]. Details in how material was prepared and quantified, as well as which cell types and developmental stages were analyzed may account for these differences. We therefore used an independent method, biochemical fractionation, to confirm that PcG proteins including PSC are associated with mitotic chromosomes (Figure 2). Our ChIP-SEQ results also demonstrate persistence of PSC on mitotic chromosomes (Figure 4).

The amount of PSC retained on mitotic chromosomes as determined by ChIP is lower than by the other methods. Based on comparisons of sequenced reads, it is only a few per cent of that bound to chromatin in control cells (Figure 4B). We cannot rule out the possibility that the condensation of chromatin reduces accessibility to the antibodies and thus decreases the signal in ChIP experiments, although H3 ChIP at PRES where PSC is lost is unchanged between control and mitotic cells (Figure 3E). Nevertheless, we think it is quite possible that much of the chromosome-associated PcG protein we detect by immunofluorescence and fractionation is not bound to specific sites. These PcG proteins may travel with mitotic chromosomes to ensure equal segregation to both new cells, or to keep to the local concentration of PcG proteins near the DNA high to facilitate rebinding after mitosis. A recent live cell imaging study of two types of cells in *Drosophila* expressing transgenic GFP-PC or GFP-PH found that only a small fraction (0.4–2%) of each of these proteins was bound in metaphase (compared with 10–20% for GFP-PC and 30–70% for GFP-PH in interphase) [30]. Interestingly, PC, and in one cell type PH, was more tightly bound in metaphase than interphase. It is possible that this tightly bound protein is the protein we detect bound to specific sites by ChIP-SEQ.

Our ChIP-SEQ data indicate that PSC binding sites can be grouped into two classes: dynamic sites that lose binding of PSC in mitosis, and persistent sites, at which these proteins are reduced but clearly still present. The dynamic sites include well-characterized PREs controlling expression of important PcG targets like the *Hox* genes. Our data thus provide clear evidence that propagation of PcG-dependent repression of most genes through mitosis does not involve persistent binding of PSC to PREs near these genes as predicted by Model 1. Our ChIP data on G2/M cells also indicates that it is unlikely that any of the PcG proteins we tested (PHO, PC, SU(Z)12) persist at *Hox* PREs in mitosis (Figure 9). In contrast, our data suggest H3K27me3 levels are unchanged in G2/M cells relative to controls, and thus that H3K27me3 is a candidate “epigenetic mark” at these sites in mitosis, as predicted in Model 2.

Based on the overlap between persistent PSC binding sites, chromatin domain borders and insulator proteins, and the finding that some of these sites flank PcG targets such as the *Hox* gene clusters (Figure 7), a possible model for the function of persistent sites is that they function as nucleation sites for re-establishment of PcG protein binding after mitosis (Figure 10). From these loading sites, PcG proteins could spread into the chromatin domain to PREs that are marked with H3K27me3 and possibly other persistent chromatin features (Figure 10). A similar model has been proposed for establishing binding of the Male Specific Lethal (MSL) dosage compensation complexes on the *Drosophila* male X-chromosome during development [52], [53]. MSL complexes are recruited to high affinity sites on the X chromosome from which they spread across the X chromosome. MSL complexes do not coat the X chromosome but preferentially spread to actively transcribed genes, at least in part through recognition of H3K36me3. MSL complexes associate stably with the X chromosome in mitosis [54], so that it is not clear if this process needs to be repeated each cell cycle.

This model predicts that the mechanisms of recruitment to persistent and dynamic sites may be different, a prediction which has not yet been tested. Perhaps interactions between insulator proteins and PcG proteins are important for binding at persistent sites; it will be interesting to determine whether insulator proteins are retained at these sites in mitosis. Recently, Van Bortle *et al.* (2012) carried out ChIP-SEQ analysis of H3K27me3 in Kc cells in which dCTCF was depleted by RNAi, and found a decrease in H3K27me3 in PcG domains in cells with depleted dCTCF [55]. This is consistent with a role for insulator proteins in maintaining PcG domains, as are earlier observations that PcG (or TrxG) mutations disrupt insulator bodies in *Drosophila* cells [56]. The paucity of long range interactions at border sites relative to other PcG sites such as those in the *Hox* clusters [19], [57], [46], [44] may contribute to the difference in PcG protein behavior at them. Perhaps disruption of long range interactions as cells enter mitosis, which might be important to allow chromatin condensation, contributes to loss of PcG proteins from sites within domains. Regions of the chromosome that are not extensively networked with distal sites (i.e. border sites) might be less disrupted by mitotic chromatin condensation, possibly allowing PcG proteins to remain bound. An alternative model for the function of persistent PSC binding sites at chromatin domain borders is that they reflect an independent role of PcG proteins in demarcating large scale chromatin domains through mitosis.

PcG proteins and their functions are widely conserved. In mammalian cells, immunofluorescence studies report some PcG protein and H3K27me3 associated with mitotic chromosomes [48], [58]. PcG proteins, including EZH2, the enzyme responsible for H3K27 methylation, are phosphorylated in a cell cycle dependent manner [59]–[64]. Cell-cycle dependent CDK-mediated phosphorylation of EZH2, which peaks in G2/M is important for maintaining PRC2 (the complex formed by EZH2) at target genes, possibly because it increases the affinity of PRC2 for non-coding RNAs. PRC2 also interacts and co-localizes with CTCF [24], [65]. Interestingly, a careful study of PcG proteins through the cell cycle in mammalian cells found that PcG bodies, which are thought to be sites of long range interactions among PcG proteins in *Drosophila* [44], are reformed in G1 although some PcG proteins and H3K27me3 persist on chromosomes through mitosis [48]. It will therefore be interesting to compare ChIP-SEQ analysis of PcG proteins in mammalian mitotic cells with the observations presented here.

While this paper was being revised, a paper describing mitotic ChIP-SEQ for the transcription factor GATA-1 in FACS-sorted mammalian cells was published [66]. Persistent GATA1 binding was observed at about 10% of interphase sites in mitosis. Degradation of GATA1 in mitosis led to slower transcriptional activation of some genes with mitotic GATA1 binding and loss of repression at other targets where GATA1 is involved in negative regulation [66]. Thus, GATA1 seems to function similarly to previously described mitotic bookmarking factors [8], [9] to ensure timely regulation of gene expression on mitotic exit. We do not know if PcG proteins carry out a bookmarking function at persistent sites, but our data indicate that at important PcG targets such as the *Hox* genes, PSC does not function as a bookmark.

In summary, we found that a key PcG protein, PSC, is lost from most sites, including well-characterized PREs, in mitosis. PSC is retained at specific sites, many of which overlap chromatin borders. We hypothesize that the persistent binding sites are important for re-establishing PcG-dependent chromatin structures and/or large scale chromatin domains after mitosis and may contribute to propagation of silencing.

## Materials and Methods

### Cell culture

*Drosophila* S2 cells (Invitrogen, Carlsbad, CA) were cultured in ESF 921 media (Expression Systems, Woodland, CA) at a density between 1 and 7×10^6^ cells/mL in shaking flasks at 27°C.

### Antibodies

The affinity-purified anti-PSC antibody raised against PSC aa 521–869 was previously described [67]. Antibodies against PC, PH, PHO and SU(Z)12 were kind gifts from J. Mueller [68]. The antibody against dRing was a gift from R. Jones [69]. The anti-CRM antibody was a gift from W. Gehring [70], and the anti-dCBP antibody was a kind gift from A. Mazo [71]. The anti-E(Z) antibody, (dL-19), and the anti-β-tubulin antibody, (d-140) were purchased from Santa Cruz Biotechnology, Inc. (Santa Cruz, CA). The anti-H3 antibodies, ChIP-grade ab1791 (for ChIP) and ab39655 (for sorting), and the anti-H3K27me3 antibody, ChIP-grade ab6002, were purchased from Abcam (Cambridge, MA). The antibody to H3Ser10p was purchased from Millipore (Billerica, MA).

### Immunofluorescence and imaging

Immunofluoresence was performed as previously described [28]. 1×10^6^ *Drosophila* S2 cells were plated on concanavalin A (0.5 mg/mL) coated coverslips in 6-well plates and allowed to attach overnight. For detergent-extraction, cells were first incubated in 1% digitonin, 20 mM HEPES, pH 7.3, 110 mM KOAc, 5 mM NaOAc, 2 mM MgOAc, 1 mM EGTA for 5 min. on ice. The rest of the staining procedure was the same for detergent-extracted and unextracted cells: cells were washed at room temperature 2× with 0.7% NaCl, incubated for 10 min. in 0.5% sodium citrate, and fixed for 8 min. in 50% methanol, 20% acetic acid. Cells were washed 5 min. in 1× PBS and permeabilized for 10 min. in PBS +1% triton-X 100. Cells were blocked for 30 min. at room temperature in 5% milk in PBS, rinsed in PBS and incubated with primary antibody diluted 1∶200 in 1% BSA+PBS overnight at 4°C. Cells were then washed 3×5 min. in PBS and incubated 2 hrs. at room temperature in 1∶200 dilution of secondary antibody in 1% BSA in PBS +0.1% Triton-X 100. Cells were washed 2×5 min. in PBS and stained 10 min. with Hoechst (0.5 µg/mL), washed 5 min. in PBS and mounted on slides.

Cells were visualized on a Zeiss LSM700 inverted confocal microscope. 0.7 µm optical sections were taken using a 63× objective. Laser power and gain were kept constant for images taken from the same slide. Images were quantified using ImageJ. A DNA mask was chosen by applying a threshold to the DNA channel using the Li method and selecting the outline of the DNA at the signal/background border. Average signal intensity for the PcG channel within this DNA mask was recorded to give PcG_DNA_, the PcG signal that overlaps DNA. A cell mask was chosen by applying a threshold to the PcG channel and selecting the outline of the cell at the signal/background border. A cytoplasmic mask was created by subtracting the DNA mask from the cell mask. Average signal intensity for the PcG channel within the cytoplasmic mask was recorded to give PcG_cyto_, the PcG signal in the cytosol. PcG_DNA_/PcG_cyto_ were calculated and averaged for at least 10 each of mitotic and interphase cells.

### Cell fractionation and chromatin isolation

Cell fractionation was carried at as in [32] with minor changes. 3.5×10^7^ asynchronously-growing, control, or colchicine-treated, G2/M, cells were treated with 2 units of DNaseI and incubated on ice for 1 hr. for total cell extract (TCE) or fractionated by resuspension to 7×10^7^ cells/mL in Buffer A (10 mM HEPES, pH 7.9, 10 mM KCl, 1.5 mM MgCl2, 0.34 M sucrose, 10% glycerol) plus 0.1% Triton-X 100, 1 mM DTT, 50 mg/mL TLCK, 10 mg/mL aprotinin, 16 mg/mL benzamidine, 10 mg/mL leupeptin, 2 mg/mL pepstatin, 10 mg/mL phenanthroline, and 0.2 mM PMSF; and incubated on ice for 5 min. The samples were centrifuged (1,300× *g*, 4 min, 4°C) to give pellet 1 (P1) and supernatant (S1). S1 was centrifuged (20,000× *g*, 15 min, 4°C) to give supernatant (S2) and pellet (P2). P1 was washed once in Buffer A and then lysed in Buffer B (3 mM EDTA, 0.2 mM EGTA, 1 mM DTT, protease inhibitors as described above). Insoluble chromatin was collected by centrifugation (1,700× *g*, 4 min, 4°C) to give pellet (P3) and supernatant (S3). P3 was washed once in Buffer B, centrifuged again under the same conditions and resuspended in SDS loading buffer and sonicated for 5 sec. with a VibraCell sonicator (Sonics & Materials, Inc., Newtown, CT) using a microtip at 25% amplitude. Fractions were run on 8% or 15% SDS PAGE gels, transferred to nitrocellulose, blotted and developed using HRP. Blots were scanned on a Typhoon Imager and quantified with ImageQuant.

### Cell synchronization and sorting

A detailed description of the synchronization and cell sorting protocol is described elsewhere [72]. Briefly, cells were treated with 350 ng/ml (880 nM) colchicine for 15 hrs., harvested and then centrifuged twice (480× *g* for 5 min.) through a 20% sucrose cushion to remove cell debris. Fixed colchicine treated or asynchronously growing S2 cells were resuspended in 0.016% Triton-X 100, 1× PBS + protease inhibitors (which are used throughout) and incubated for 15 min. on ice. Cells were washed in 1% BSA, 0.1% Triton-X 100, 1× PBS and incubated with 2.7 µg/mL FITC-conjugated α-H3Ser10p antibody (colchicine-treated cells) or 3 µg/mL α-H3 antibody (control cells) at a concentration of 1×10^7^ cells/mL and incubated on ice in the dark for 30 min. Cells were washed with 1% BSA, 0.1% Triton-X 100, 1× PBS. Control cells were incubated with FITC-conjugated secondary antibody for 30 min. and washed with 1% BSA, 0.1% Triton-X 100, 1× PBS. Cells were resuspended in 1× PBS, 10% horse serum and incubated overnight at 4°C in the dark. Cells were passed through a 40 µM filter and sorted by the FAS Center for Systems Biology Flow Cytometry Core on a MoFlo Legacy Cell Sorter (Beckman Coulter) to collect FITC-positive cells. Pre- and post-sorted cell populations were analyzed on an LSRII cell sorter (BD Biosciences).

### Chromatin immunoprecipitation (ChIP)

ChIP was carried out as previously described [67]. Briefly, *Drosophila* S2 cells were fixed in 1% formaldehyde and quenched with 0.125 mM glycine, pH 7. Cells were washed with 1× PBS, wash buffer I (10 mM Hepes, pH 7.6, 10 mM EDTA, 0.5 mM EGTA, 0.25% Triton X-100, plus protease inhibitors as described above), and wash buffer II (10 mM Hepes, pH 7.6, 200 mM NaCl, 1 mM EDTA, 0.5 mM EGTA, 0.01% Triton X-100, plus protease inhibitors as described above). Cells were centrifuged (250× *g*, 4 min, 4°C) and resuspended in sonication buffer (50 mM HEPES, pH 7.5, 500 mM NaCl, 1 mM EDTA, 1% Triton X-100, 0.1% sodium deoxycholate, 0.1% SDS) to a cell concentration of 2×10^7^ cells/mL in 2 mL and sonicated with 10×30 second pulses with 30 seconds between pulses using a Sonics Vibracell sonicator at 40% power. Following sonication, samples were centrifuged for 5 min. at full speed in a refrigerated microcentrifuge. The supernatant was used for ChIP. 100 µL of chromatin (corresponding to ∼2×10^6^ cells) were used for each reaction and were adjusted to 1× ChIP binding buffer (15 mM Tris, pH 8, 150 mM NaCl, 1 mM EDTA, 1% Triton X-100, 0.01% SDS). Protein A-agarose-ChIP was carried out as previously described [67]. Biotin-ChIP was carried out as follows: samples were pre-cleared with magnetic Dynabeads M-280 streptavidin (Invitrogen) blocked with 0.2 mg/mL salmon sperm DNA. Antibodies were biotinylated using a kit according to the manufacturer's instructions (Anaspec or Thermo-Pierce). 4 µg of biotinylated antibody was used, which in titration experiments was shown to be saturating, and 0.5 µg of anti-H3 was used and samples were incubated overnight. Magnetic beads were added to capture immune complexes for 2 hours at 4°C. Magnetic beads were washed 3× in ChIP binding buffer for 5 min. at RT and were isolated on a magnetic rack for 2 min. DNA was eluted by addition of biotin elution buffer (1% SDS, 0.5 M NaCl). Elutions were incubated 6 hours at 65°C to reverse crosslinks. Samples were treated for 30 min. with RNase A at 37°C and 1 hour with Proteinase K at 55°C, and DNA was purified with a Nucleospin Extract II kit (Macherey-Nagel). qPCR was carried out on a Bio-Rad IQ5 machine using SYBR green (Bio-Rad).

### Genome-wide sequencing and data analysis

6–50 ng of input or immunoprecipitated DNA were submitted to the BioMicro Center (MIT, Cambridge, MA) for library generation and sequencing on an Illumina HiSeq 2000. Two sets of samples were first fragmented using a Bioruptor 300 (Diagenode). 25 ng of DNA was biorupted for 80 pulses on the low setting to generate fragments ∼180 bp. 36-bp reads were aligned to the *D. melanogaster* genome (dm3) using Bowtie 1.1.2 [73] through Galaxy [74]–[76] retaining uniquely mapping reads with up to 2 mismatches in the first 28 bp. Binding sites were identified using MACS through Galaxy using fragment size 36, genome size 120,000,000, bandwidth 200, cutoff P-value<10^−5^. The results were filtered to retain peaks with a false discovery rate of 5% or below for all samples except for mitotic PH. Peaks on chromosome U and Uextra were removed. Pearson's correlation coefficient was used as a measure of correlation between datasets and was computed for number of reads within each peak for all called peaks in either of the sets. Heatmap distributions were generated using seqMINER 1.2.1 [77] and visualized using GiTools [78]. Average profile plots and distribution analysis were done with CEAS 1.0.2 [79]. Promoter regions were defined as 250 bp upstream of the TSS. Raw and processed files are available at GEO (http://www.ncbi.nlm.nih.gov/geo/) under the accession number GSE38166.

### Comparison with other datasets

ChIP-chip tiling array data and ChIP-seq data were downloaded from the modENCODE consortium (modMine.org), GEO (www.ncbi.nlm.nih.gov/geo/), or supplemental material as available [80], [44]. Called peaks from the original studies were used to retain consistency with published work where possible. Overlaps were calculated using BEDtools 2.12.0.

## Supporting Information

Figure S1 Genome-wide binding of PH in control cells overlaps extensively with PSC. A) Schematic of the constructs integrated into S2 cells. Both the BLRP-FLAG-PH construct and the biotin ligase BirA construct are controlled by the metallothione promoter (MT_pro_). The BirA construct additionally contains the puromycin gene which was used for slelection [81]. B) Pulldown of BLRP-PH with streptavidin-coated beads from the PH S2 cell line or WT S2 cell line indicates that PH is biotin-tagged. C) Western blot shows that the level of expression of PH in the PH S2 cells is comparable to the level of expression in WT S2 cells. D) Venn diagram of overlap of peaks for PSC and PH from H3-sorted control cells shows a high degree of overlap for these datasets. E) Normalized read density in 50 bp windows of PH (black line) and PSC (gray line) in control cells averaged over control PH binding sites (left panel) or control PSC binding sites (right panel). F) Sequence tracks from ChIP-SEQ comparing PH binding (top track, black) and PSC binding (bottom track, gray, same as in Figure 4C) in control cells over the BX-C and the *engrailed* locus. Y-axis is normalized reads per million (RPM)/10 bp. Chromosome position and gene models are shown at the bottom.
(TIF)

Figure S2 PSC distribution on chromosomes in mitosis. A) Distribution of PSC binding sites across chromosomes in control (top panel) and mitotic (bottom panel) cells. X-axis is chromosomal position and Y-axis is peak height given by relative sequence reads. The peak height is given by normalized relative sequence reads. B) Quantification of PSC peaks per chromosome shows the percentage of peaks per chromosome remains the same, except for chromosome 4, where all peaks are lost. C) Plot of difference in % of binding sites per 5 Mbp window between control and mitotic distributions for PSC across chromosomes. The locations of the ANT-C and BX-C on chromosome 3R are indicated.
(TIF)

Table S1 Genes bound by PSC in mitosis (within 2 kb of TSS). List of genes with PSC bound in mitosis.
(XLS)

Table S2 Genes bound by PSC in mitosis with transcription-related GO terms. Subset of genes with PSC bound in mitosis that have transcription-related GO terms that are enriched versus genomic background.
(XLS)

Table S3 Genes bound by PSC in mitosis with cell cycle-related GO terms. Subset of genes with PSC bound in mitosis that have cell-cycle related GO terms that are enriched versus genomic background and versus the control as background.
(XLS)

## References

[1] Dovat S, Ronni T, Russell D, Ferrini R, Cobb BS, et al (2002) A common mechanism for mitotic inactivation of C2H2 zinc finger DNA-binding domains. Genes Dev 16 (23) 2985–2990. 12464629 10.1101/gad.1040502 PMC187490

[2] Fischle W, Tseng BS, Dormann HL, Ueberheide BM, Garcia BA, et al (2005) Regulation of HP1-chromatin binding by histone H3 methylation and phosphorylation. Nature 438 (7071) 1116–1122. 16222246 10.1038/nature04219

[3] Hirota T, Lipp JJ, Toh BH, Peters JM (2005) Histone H3 serine 10 phosphorylation by Aurora B causes HP1 dissociation from heterochromatin. Nature 438 (7071) 1176–1180. 16222244 10.1038/nature04254

[4] Xing H, Wilkerson DC, Mayhew CN, Lubert EJ, Skaggs HS, et al (2005) Mechanism of hsp70i gene bookmarking. Science 307 (5708) 421–423. 15662014 10.1126/science.1106478

[5] Delcuve GP, He S, Davie JR (2008) Mitotic partitioning of transcription factors. J Cell Biochem 105 (1) 1–8. 18459122 10.1002/jcb.21806

[6] Xing H, Vanderford NL, Sarge KD (2008) The TBP-PP2A mitotic complex bookmarks genes by preventing condensin action. Nat Cell Biol 10 (11) 1318–1323. 18931662 10.1038/ncb1790 PMC2577711

[7] Blobel GA, Kadauke S, Wang E, Lau AW, Zuber J, et al (2009) A reconfigured pattern of MLL occupancy within mitotic chromatin promotes rapid transcriptional reactivation following mitotic exit. Mol Cell 36 (6) 970–983. 20064463 10.1016/j.molcel.2009.12.001 PMC2818742

[8] Zaidi SK, Young DW, Montecino M, van Wijnen AJ, Stein JL, et al (2011) Bookmarking the genome: maintenance of epigenetic information. J Biol Chem 286 (21) 18355–18361. 21454629 10.1074/jbc.R110.197061 PMC3099651

[9] Zhao R, Nakamura T, Fu Y, Lazar Z, Spector DL (2011) Gene bookmarking accelerates the kinetics of post-mitotic transcriptional re-activation. Nat Cell Biol 13 (11) 1295–1304. 21983563 10.1038/ncb2341 PMC3210065

[10] Pirrotta V (1998) Polycombing the genome: PcG, trxG, and chromatin silencing. Cell 93: 333–336. 9590168 10.1016/s0092-8674(00)81162-9

[11] Simon JA, Tamkun JW (2002) Programming off and on states in chromatin: mechanisms of Polycomb and trithorax group complexes. Curr Opin Genet Dev 12 (2) 210–218. 11893495 10.1016/s0959-437x(02)00288-5

[12] Ringrose L, Paro R (2004) Epigenetic regulation of cellular memory by the Polycomb and Trithorax group proteins. Annu Rev Genet 38: 413–443. 15568982 10.1146/annurev.genet.38.072902.091907

[13] Grimaud C, Negre N, Cavalli G (2006) From genetics to epigenetics: the tale of Polycomb group and trithorax group genes. Chromosome Res 14 (4) 363–375. 16821133 10.1007/s10577-006-1069-y

[14] Schuettengruber B, Chourrout D, Vervoort M, Leblanc B, Cavalli G (2007) Genome regulation by polycomb and trithorax proteins. Cell 128 (4) 735–745. 17320510 10.1016/j.cell.2007.02.009

[15] Richly H, Aloia L, Di Croce L (2011) Roles of the Polycomb group proteins in stem cells and cancer. Cell Death Dis 2: e204. 21881606 10.1038/cddis.2011.84 PMC3186902

[16] Muller J, Verrijzer P (2009) Biochemical mechanisms of gene regulation by polycomb group protein complexes. Curr Opin Genet Dev 19 (2) 150–158. 19345089 10.1016/j.gde.2009.03.001

[17] Simon JA, Kingston RE (2009) Mechanisms of polycomb gene silencing: knowns and unknowns. Nat Rev Mol Cell Biol 10 (10) 697–708. 19738629 10.1038/nrm2763

[18] Muller J, Kassis JA (2006) Polycomb response elements and targeting of Polycomb group proteins in Drosophila. Curr Opin Genet Dev 16 (5) 476–484. 16914306 10.1016/j.gde.2006.08.005

[19] Lanzuolo C, Roure V, Dekker J, Bantignies F, Orlando V (2007) Polycomb response elements mediate the formation of chromosome higher-order structures in the bithorax complex. Nat Cell Biol 9 (10) 1167–1174. 17828248 10.1038/ncb1637

[20] Bantignies F, Cavalli G (2011) Polycomb group proteins: repression in 3D. Trends Genet 27: 454–464. 21794944 10.1016/j.tig.2011.06.008

[21] Moshkin YM, Alekseyenko AA, Semeshin VF, Spierer A, Spierer P, et al (2001) The Bithorax complex of Drosophile melanogaster: underreplication and morphology in polytene chromosomes. Proc Natl Acad Sci USA 98: 570–574. 11136231 10.1073/pnas.021353598 PMC14628

[22] Comet I, Schuettengruber B, Sexton T, Cavalli G (2011) A chromatin insulator driving three-dimensional Polycomb response element (PRE) contacts and Polycomb association with the chromatin fiber. Proc Natl Acad Sci U S A 108 (6) 2294–2299. 21262819 10.1073/pnas.1002059108 PMC3038747

[23] Li HB, Muller M, Bahechar IA, Kyrchanova O, Ohno K, et al (2011) Insulators, not Polycomb response elements, are required for long-range interactions between Polycomb targets in Drosophila melanogaster. Mol Cell Biol 31 (4) 616–625. 21135119 10.1128/MCB.00849-10 PMC3028641

[24] Pirrotta V, Li HB (2012) A view of nuclear Polycomb bodies. Curr Opin Genet Dev 22 (2) 101–109. 22178420 10.1016/j.gde.2011.11.004 PMC3329586

[25] Yang J, Corces VG (2012) Insulators, long-range interactions, and genome function. Curr Opin Genet Dev 22 (2) 86–92. 22265227 10.1016/j.gde.2011.12.007 PMC3337973

[26] Francis NJ, Kingston RE (2001) Mechanisms of transcriptional memory. Nat Rev Mol Cell Biol 2 (6) 409–421. 11389465 10.1038/35073039

[27] Buchenau P, Hodgson J, Strutt H, Arndt-Jovin DJ (1998) The distribution of Polycomb-group proteins during cell division and development in *Drosophila* embryos: impact on models for silencing. J Cell Biol 141 (2) 469–481. 9548724 10.1083/jcb.141.2.469 PMC2148446

[28] Fanti L, Perrini B, Piacentini L, Berloco M, Marchetti E, et al (2008) The trithorax group and Pc group proteins are differentially involved in heterochromatin formation in Drosophila. Chromosoma 117 (1) 25–39. 17823810 10.1007/s00412-007-0123-7

[29] Beck SA, Falconer E, Catching A, Hodgson JW, Brock HW (2010) Cell cycle defects in polyhomeotic mutants are caused by abrogation of the DNA damage checkpoint. Dev Biol 339 (2) 320–328. 20045683 10.1016/j.ydbio.2009.12.031

[30] Fonseca JP, Steffen PA, Muller S, Lu J, Sawicka A, et al (2012) In vivo Polycomb kinetics and mitotic chromatin binding distinguish stem cells from differentiated cells. Genes Dev 26 (8) 857–871. 22508729 10.1101/gad.184648.111 PMC3337459

[31] Rowbotham SP, Barki L, Neves-Costa A, Santos F, Dean W, et al (2011) Maintenance of silent chromatin through replication requires SWI/SNF-like chromatin remodeler SMARCAD1. Mol Cell 42 (3) 285–296. 21549307 10.1016/j.molcel.2011.02.036

[32] Mendez J, Stillman B (2000) Chromatin association of human origin recognition complex, cdc6, and minichromosome maintenance proteins during the cell cycle: assembly of prereplication complexes in late mitosis. Mol Cell Biol 20 (22) 8602–8612. 11046155 10.1128/mcb.20.22.8602-8612.2000 PMC102165

[33] Kouskouti A, Talianidis I (2005) Histone modifications defining active genes persist after transcriptional and mitotic inactivation. Embo J 24 (2) 347–357. 15616580 10.1038/sj.emboj.7600516 PMC545808

[34] Zhang Y, Liu T, Meyer CA, Eeckhoute J, Johnson DS, et al (2008) Model-based analysis of ChIP-Seq (MACS). Genome Biol 9 (9) R137. 18798982 10.1186/gb-2008-9-9-r137 PMC2592715

[35] Rastelli L, Chan CS, Pirrotta V (1993) Related chromosome binding sites for zeste, suppressors of zeste and Polycomb group proteins in *Drosophila* and their dependence on Enhancer of zeste function. EMBO J 12: 1513–1522. 8467801 10.1002/j.1460-2075.1993.tb05795.x PMC413364

[36] Beisel C, Buness A, Roustan-Espinosa IM, Koch B, Schmitt S, et al (2007) Comparing active and repressed expression states of genes controlled by the Polycomb/Trithorax group proteins. Proc Natl Acad Sci U S A 104 (42) 16615–16620. 17921257 10.1073/pnas.0701538104 PMC2001164

[37] Enderle D, Beisel C, Stadler MB, Gerstung M, Athri P, et al (2011) Polycomb preferentially targets stalled promoters of coding and noncoding transcripts. Genome Res 21 (2) 216–226. 21177970 10.1101/gr.114348.110 PMC3032925

[38] Ho JW, Bishop E, Karchenko PV, Negre N, White KP, et al (2011) ChIP-chip versus ChIP-seq: lessons for experimental design and data analysis. BMC Genomics 12: 134. 21356108 10.1186/1471-2164-12-134 PMC3053263

[39] Consortium m (2010) Identification of functional elements and regulatory circuits by Drosophila modENCODE. Science 330 (6012) 1787–1797. 21177974 10.1126/science.1198374 PMC3192495

[40] Kharchenko PV, Alekseyenko AA, Schwartz YB, Minoda A, Riddle NC, et al (2010) Comprehensive analysis of the chromatin landscape in Drosophila melanogaster. Nature 471 (7339) 480–485. 21179089 10.1038/nature09725 PMC3109908

[41] Huang da W, Sherman BT, Lempicki RA (2009) Bioinformatics enrichment tools: paths toward the comprehensive functional analysis of large gene lists. Nucleic Acids Res 37 (1) 1–13. 19033363 10.1093/nar/gkn923 PMC2615629

[42] Huang da W, Sherman BT, Lempicki RA (2009) Systematic and integrative analysis of large gene lists using DAVID bioinformatics resources. Nat Protoc 4 (1) 44–57. 19131956 10.1038/nprot.2008.211

[43] Wood AM, Van Bortle K, Ramos E, Takenaka N, Rohrbaugh M, et al (2011) Regulation of chromatin organization and inducible gene expression by a Drosophila insulator. Mol Cell 44 (1) 29–38. 21981916 10.1016/j.molcel.2011.07.035 PMC3190163

[44] Sexton T, Yaffe E, Kenigsberg E, Bantignies F, Leblanc B, et al (2012) Three-Dimensional Folding and Functional Organization Principles of the Drosophila Genome. Cell 10.1016/j.cell.2012.01.010 22265598

[45] Lanzuolo C, Lo Sardo F, Diamantini A, Orlando V (2011) PcG complexes set the stage for epigenetic inheritance of gene silencing in early S phase before replication. PLoS Genet 7: e1002370 doi:10.1371/journal.pgen.1002370. 22072989 10.1371/journal.pgen.1002370 PMC3207895

[46] Tolhuis B, Blom M, Kerkhoven RM, Pagie L, Teunissen H, et al (2011) Interactions among Polycomb domains are guided by chromosome architecture. PLoS Genet 7: e1001343 doi:10.1371/journal.pgen.1001343. 21455484 10.1371/journal.pgen.1001343 PMC3063757

[47] Goto H, Tomono Y, Ajiro K, Kosako H, Fujita M, et al (1999) Identification of a novel phosphorylation site on histone H3 coupled with mitotic chromosome condensation. J Biol Chem 274 (36) 25543–25549. 10464286 10.1074/jbc.274.36.25543

[48] Aoto T, Saitoh N, Sakamoto Y, Watanabe S, Nakao M (2008) Polycomb group protein-associated chromatin is reproduced in post-mitotic G1 phase and is required for S phase progression. J Biol Chem 283 (27) 18905–18915. 18453536 10.1074/jbc.M709322200

[49] Li Z, Tatsuke T, Sakashita K, Zhu L, Xu J, et al (2012) Identification and characterization of Polycomb group genes in the silkworm, Bombyx mori. Mol Biol Rep 39 (5) 5575–5588. 22187347 10.1007/s11033-011-1362-5

[50] Petruk S, Sedkov Y, Johnston DM, Hodgson JW, Black KL, et al (2012) TrxG and PcG Proteins but Not Methylated Histones Remain Associated with DNA through Replication. Cell 150 (5) 922–933. 22921915 10.1016/j.cell.2012.06.046 PMC3432699

[51] Martin EC, Adler PN (1993) The Polycomb group gene Posterior Sex Combs encodes a chromosomal protein. Development 117: 641–655. 7687213 10.1242/dev.117.2.641

[52] Sural TH, Peng S, Li B, Workman JL, Park PJ, et al (2008) The MSL3 chromodomain directs a key targeting step for dosage compensation of the Drosophila melanogaster X chromosome. Nat Struct Mol Biol 15 (12) 1318–1325. 19029895 10.1038/nsmb.1520 PMC2636508

[53] Gelbart ME, Kuroda MI (2009) Drosophila dosage compensation: a complex voyage to the X chromosome. Development 136 (9) 1399–1410. 19363150 10.1242/dev.029645 PMC2674252

[54] Strukov YG, Sural TH, Kuroda MI, Sedat JW (2011) Evidence of activity-specific, radial organization of mitotic chromosomes in Drosophila. PLoS Biol 9: e1000574 doi:10.1371/journal.pbio.1000574. 21264350 10.1371/journal.pbio.1000574 PMC3019107

[55] Van Bortle K, Ramos E, Takenaka N, Yang J, Wahi J, et al (2012) Drosophila CTCF tandemly aligns with other insulator proteins at the borders of H3K27me3 domains. Genome Res 10.1101/gr.136788.111 PMC3483547 22722341

[56] Gerasimova TI, Corces VG (1998) Polycomb and trithorax group proteins mediate the function of a chromatin insulator. Cell 92 (4) 511–521. 9491892 10.1016/s0092-8674(00)80944-7

[57] Bantignies F, Roure V, Comet I, Leblanc B, Schuettengruber B, et al (2011) Polycomb-dependent regulatory contacts between distant Hox loci in Drosophila. Cell 144 (2) 214–226. 21241892 10.1016/j.cell.2010.12.026

[58] Hansen KH, Bracken AP, Pasini D, Dietrich N, Gehani SS, et al (2008) A model for transmission of the H3K27me3 epigenetic mark. Nat Cell Biol 10 (11) 1291–1300. 18931660 10.1038/ncb1787

[59] Voncken JW, Schweizer D, Aagaard L, Sattler L, Jantsch MF, et al (1999) Chromatin-association of the Polycomb group protein BMI1 is cell cycle-regulated and correlates with its phosphorylation status. J Cell Sci 112 (Pt 24) 4627–4639. 10574711 10.1242/jcs.112.24.4627

[60] Chen S, Bohrer LR, Rai AN, Pan Y, Gan L, et al (2010) Cyclin-dependent kinases regulate epigenetic gene silencing through phosphorylation of EZH2. Nat Cell Biol 12 (11) 1108–1114. 20935635 10.1038/ncb2116 PMC3292434

[61] Kaneko S, Li G, Son J, Xu CF, Margueron R, et al (2010) Phosphorylation of the PRC2 component Ezh2 is cell cycle-regulated and up-regulates its binding to ncRNA. Genes Dev 24 (23) 2615–2620. 21123648 10.1101/gad.1983810 PMC2994035

[62] Wei Y, Chen YH, Li LY, Lang J, Yeh SP, et al (2011) CDK1-dependent phosphorylation of EZH2 suppresses methylation of H3K27 and promotes osteogenic differentiation of human mesenchymal stem cells. Nat Cell Biol 13 (1) 87–94. 21131960 10.1038/ncb2139 PMC3076036

[63] Wu SC, Zhang Y (2011) Cyclin-dependent kinase 1 (CDK1)-mediated phosphorylation of enhancer of zeste 2 (Ezh2) regulates its stability. J Biol Chem 286 (32) 28511–28519. 21659531 10.1074/jbc.M111.240515 PMC3151093

[64] Zeng X, Chen S, Huang H (2011) Phosphorylation of EZH2 by CDK1 and CDK2: a possible regulatory mechanism of transmission of the H3K27me3 epigenetic mark through cell divisions. Cell Cycle 10 (4) 579–583. 21278485 10.4161/cc.10.4.14722 PMC3174000

[65] Zhang H, Niu B, Hu JF, Ge S, Wang H, et al (2011) Interruption of intrachromosomal looping by CCCTC binding factor decoy proteins abrogates genomic imprinting of human insulin-like growth factor II. J Cell Biol 193 (3) 475–487. 21536749 10.1083/jcb.201101021 PMC3087012

[66] Kadauke S, Udugama MI, Pawlicki JM, Achtman JC, Jain DP, et al (2012) Tissue-Specific Mitotic Bookmarking by Hematopoietic Transcription Factor GATA1. Cell 150 (4) 725–737. 22901805 10.1016/j.cell.2012.06.038 PMC3425057

[67] Francis NJ, Follmer NE, Simon MD, Aghia G, Butler JD (2009) Polycomb Proteins Remain Bound to Chromatin and DNA during DNA Replication In Vitro. Cell 110–122. 10.1016/j.cell.2009.02.017 PMC2667909 19303136

[68] Papp B, Muller J (2006) Histone trimethylation and the maintenance of transcriptional ON and OFF states by trxG and PcG proteins. Genes Dev 20 (15) 2041–2054. 16882982 10.1101/gad.388706 PMC1536056

[69] Wang L, Brown JL, Cao R, Zhang Y, Kassis JA, et al (2004) Hierarchical recruitment of polycomb group silencing complexes. Mol Cell 14: 637–646. 15175158 10.1016/j.molcel.2004.05.009

[70] Yamamoto Y, Girard F, Bello B, Affolter M, Gehring WJ (1997) The *cramped* gene of Drosophila is a member of the *Polycomb*-group, and interacts with *mus209*, the gene encoding Proliferating Cell Nuclear Antigen. Development 124: 3385–3394. 9310333 10.1242/dev.124.17.3385

[71] Petruk S, Sedkov Y, Smith S, Tillib S, Kraevski V, et al (2001) Trithorax and dCBP acting in a complex to maintain expression of a homeotic gene. Science 294: 1331–1334. 11701926 10.1126/science.1065683

[72] Follmer NE, Francis NJ (accepted for publication) Preparation of Drosophila tissue culture cells from different stages of the cell cycle for chromatin immunoprecipitation using centrifugal counterflow elutriation and fluorescence-activate cell sorting. Methods in Enzymology 10.1016/B978-0-12-391938-0.00011-2 22929773

[73] Langmead B, Trapnell C, Pop M, Salzberg SL (2009) Ultrafast and memory-efficient alignment of short DNA sequences to the human genome. Genome Biol 10 (3) R25. 19261174 10.1186/gb-2009-10-3-r25 PMC2690996

[74] Giardine B, Riemer C, Hardison RC, Burhans R, Elnitski L, et al (2005) Galaxy: a platform for interactive large-scale genome analysis. Genome Res 15 (10) 1451–1455. 16169926 10.1101/gr.4086505 PMC1240089

[75] Blankenberg D, Von Kuster G, Coraor N, Ananda G, Lazarus R, et al (2010) Galaxy: a web-based genome analysis tool for experimentalists. Curr Protoc Mol Biol Chapter 19: Unit 19 10 11–21. 10.1002/0471142727.mb1910s89 PMC4264107 20069535

[76] Goecks J, Nekrutenko A, Taylor J (2010) Galaxy: a comprehensive approach for supporting accessible, reproducible, and transparent computational research in the life sciences. Genome Biol 11 (8) R86. 20738864 10.1186/gb-2010-11-8-r86 PMC2945788

[77] Ye T, Krebs AR, Choukrallah MA, Keime C, Plewniak F, et al (2011) seqMINER: an integrated ChIP-seq data interpretation platform. Nucleic Acids Res 39 (6) e35. 21177645 10.1093/nar/gkq1287 PMC3064796

[78] Perez-Llamas C, Lopez-Bigas N (2011) Gitools: analysis and visualisation of genomic data using interactive heat-maps. PLoS ONE 6: e19541 doi:10.1371/journal.pone.0019541. 21602921 10.1371/journal.pone.0019541 PMC3094337

[79] Shin H, Liu T, Manrai AK, Liu XS (2009) CEAS: cis-regulatory element annotation system. Bioinformatics 25 (19) 2605–2606. 19689956 10.1093/bioinformatics/btp479

[80] Schwartz YB, Kahn TG, Nix DA, Li XY, Bourgon R, et al (2006) Genome-wide analysis of Polycomb targets in Drosophila melanogaster. Nat Genet 38 (6) 700–705. 16732288 10.1038/ng1817

[81] Mito Y, Henikoff JG, Henikoff S (2005) Genome-scale profiling of histone H3.3 replacement patterns. Nat Genet 37 (10) 1090–1097. 16155569 10.1038/ng1637

