# Supplementary figures and images for: A Polycomb Group Protein Is Retained at Specific Sites on Chromatin in Mitosis
Source: PLoS Genet. 2012 Dec 20;8(12):e1003135. doi: 10.1371/journal.pgen.1003135 (PMC3527277; doi:10.1371/journal.pgen.1003135)

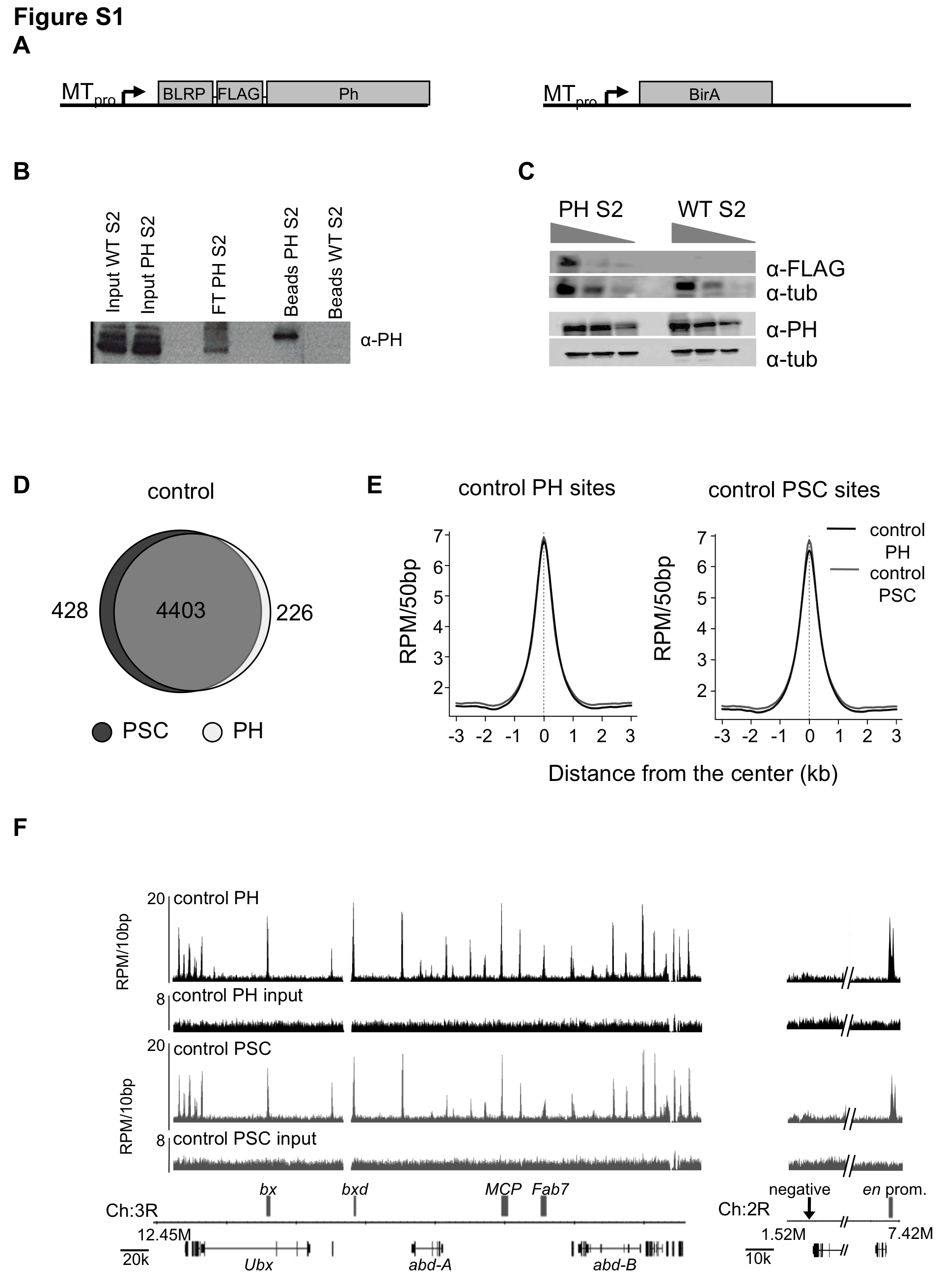

Supplement: Figure S1 — Genome-wide binding of PH in control cells overlaps extensively with PSC. A) Schematic of the constructs integrated into S2 cells. Both the BLRP-FLAG-PH construct and the biotin ligase BirA construct are controlled by the metallothione promoter (MTpro). The BirA construct additionally contains the puromycin gene which was used for slelection [81]. B) Pulldown of BLRP-PH with streptavidin-coated beads from the PH S2 cell line or WT S2 cell line indicates that PH is biotin-tagged. C) Western blot shows that the level of expression of PH in the PH S2 cells is comparable to the level of expression in WT S2 cells. D) Venn diagram of overlap of peaks for PSC and PH from H3-sorted control cells shows a high degree of overlap for these datasets. E) Normalized read density in 50 bp windows of PH (black line) and PSC (gray line) in control cells averaged over control PH binding sites (left panel) or control PSC binding sites (right panel). F) Sequence tracks from ChIP-SEQ comparing PH binding (top track, black) and PSC binding (bottom track, gray, same as in Figure 4C) in control cells over the BX-C and the engrailed locus. Y-axis is normalized reads per million (RPM)/10 bp. Chromosome position and gene models are shown at the bottom. (TIF) [file pgen.1003135.s001.tif]

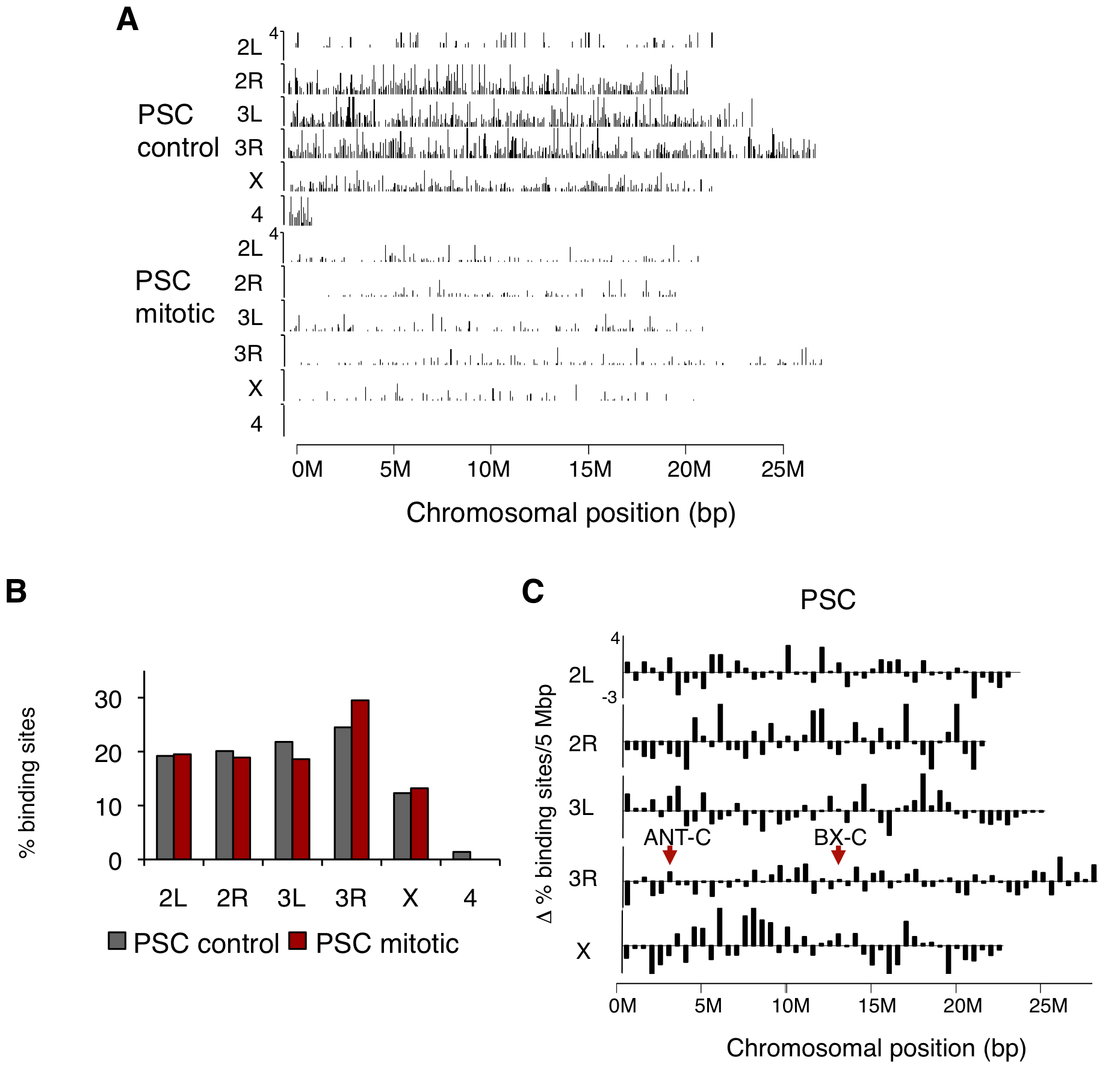

Supplement: Figure S2 — PSC distribution on chromosomes in mitosis. A) Distribution of PSC binding sites across chromosomes in control (top panel) and mitotic (bottom panel) cells. X-axis is chromosomal position and Y-axis is peak height given by relative sequence reads. The peak height is given by normalized relative sequence reads. B) Quantification of PSC peaks per chromosome shows the percentage of peaks per chromosome remains the same, except for chromosome 4, where all peaks are lost. C) Plot of difference in % of binding sites per 5 Mbp window between control and mitotic distributions for PSC across chromosomes. The locations of the ANT-C and BX-C on chromosome 3R are indicated. (TIF) [file pgen.1003135.s002.tif]
